# Current and past climate co‐shape community‐level plant species richness in the Western Siberian Arctic

**DOI:** 10.1002/ece3.11140

**Published:** 2024-03-17

**Authors:** Vitalii Zemlianskii, Philipp Brun, Niklaus E. Zimmermann, Ksenia Ermokhina, Olga Khitun, Natalia Koroleva, Gabriela Schaepman‐Strub

**Affiliations:** ^1^ Department of Evolutionary Biology and Environmental Studies UZH Zurich Switzerland; ^2^ Landscape Dynamics Research Unit, Swiss Federal Institute for Forest Snow and Landscape Research WSL Birmensdorf Switzerland; ^3^ A.N. Severtsov Institute of Ecology and Evolution Russian Academy of Sciences Moscow Russia; ^4^ Komarov Botanical Institute Russian Academy of Science Saint Petersburg Russia; ^5^ Polar‐Alpine Botanical Garden Russian Academy of Science Kirovsk Russia

**Keywords:** anthropogenic impact, Arctic vegetation, Arctic vegetation archive, community‐level plant species richness, macroecological modeling, paleoclimate predictors

## Abstract

The Arctic ecosystems and their species are exposed to amplified climate warming and, in some regions, to rapidly developing economic activities. This study assesses, models, and maps the geographic patterns of community‐level plant species richness in the Western Siberian Arctic and estimates the relative impact of environmental and anthropogenic factors driving these patterns. With our study, we aim at contributing toward conservation efforts for Arctic plant diversity in the Western Siberian Arctic. Western Siberian Arctic, Russia. We investigated the relative importance of environmental and anthropogenic predictors of community‐level plant species richness in the Western Siberian Arctic using macroecological models trained with an extensive geobotanical dataset. We included vascular plants, mosses and lichens in our analysis, as non‐vascular plants substantially contribute to species richness and ecosystem functions in the Arctic. We found that the mean community‐level plant species richness in this vast Arctic region does not decrease with increasing latitude. Instead, we identified an increase in species richness from South‐West to North‐East, which can be well explained by environmental factors. We found that paleoclimatic factors exhibit higher explained deviance compared to contemporary climate predictors, potentially indicating a lasting impact of ancient climate on tundra plant species richness. We also show that the existing protected areas cover only a small fraction of the regions with highest species richness. Our results reveal complex spatial patterns of community‐level species richness in the Western Siberian Arctic. We show that climatic factors such as temperature (including paleotemperature) and precipitation are the main drivers of plant species richness in this area, and the role of relief is clearly secondary. We suggest that while community‐level plant species richness is mostly driven by environmental factors, an improved spatial sampling will be needed to robustly and more precisely assess the impact of human activities on community‐level species richness patterns. Our approach and results can be used to design conservation strategies and to investigate drivers of plant species richness in other arctic regions.

## Graphical abstract

We investigated the relative impact of environmental and anthropogenic factors on community‐level plant species richness in the Western Siberian Arctic using macroecological models. Our results reveal complex spatial patterns of community‐level species richness in the Western Siberian Arctic. We show that climatic factors such as temperature (including paleotemperature) and precipitation are the main drivers of plant species richness in this area, and the role of relief is clearly secondary. We suggest that while community‐level plant species richness is mostly driven by environmental factors, an improved spatial sampling is needed to robustly and more precisely assess the impact of human activities on community‐level species richness patterns.

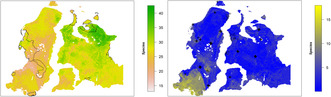

## 1 INTRODUCTION

The documentation of Arctic plant diversity and its distribution under global change is one of the key priorities of international science and policy agendas as coordinated by the Conservation of Arctic Flora and Fauna (CAFF, 1997) of the Arctic Council and the International Arctic Science Committee (IASC). This information is urgently needed for the identification of Arctic biodiversity hotspots, which are a major target for nature protection and conservation (UN Convention on Biological diversity) (СBD, 1992). Plant diversity in the Arctic is usually studied at regional (hundreds of square kilometers), local (square kilometers), and community (square meters) levels. Despite Arctic regional and (to a lesser extent) local plant diversity being relatively well documented, the community‐level distribution of plant diversity across broad spatial extents and its drivers remains understudied, especially in the Siberian part of the Arctic (Daniëls et al., 2005, 2013; Khitun et al., 2016; Walker et al., 1994). Yet, the immediate scale at which plant diversity drives ecosystem processes and responds to environmental change is the community scale. Understanding the distribution of plant diversity and its relation to environmental and anthropogenic drivers at the community level is therefore key, especially in regions exposed to amplified global change such as the Arctic.

Species richness across plant communities in the Arctic is strongly related to local abiotic factors, such as soil moisture, meso‐ and microrelief, wind speed and exposure, permafrost, and soil conditions (Iturrate‐Garcia et al., 2016; Schultz, 2005; Walker et al., 2019), which can promote high heterogeneity among communities at small spatial scales. This heterogeneity is often larger than inter‐regional differences between communities belonging to the same vegetation type (Khitun, 1998; Khitun & Rebristaya, 1998). Furthermore, anthropogenic factors play an increasingly important role in shaping Arctic vegetation, changing community composition, threatening some local species (especially, lichens and mosses), and simultaneously increasing total plant species richness through introduction of new species and habitat change (Arefev et al., 2010; Daniëls et al., 2013; Forbes, 1995, 1997; Nellemann et al., 2001; Povoroznyuk et al., 2022; Rebristaya & Khitun, 1998).

The Western Siberian tundra is a rapidly transforming region of the Arctic (Kozlova, 2013; Kumpula et al., 2011, 2012; Walker et al., 2012). The combination of multiple interacting factors including climate change, infrastructure expansion, fossil fuel extraction (Skipin et al., 2014), reindeer pressure (Egelkraut et al., 2020; Kryazhimskii et al., 2011; Veselkin et al., 2021), and species invasions, contributes to large‐scale ecosystem degradation within and beyond areas directly affected by economic activity (Forbes et al., 2009; Golovatin et al., 2012). The high landscape homogeneity (Rebristaya, 2013) and the large extent (about 300,000 km^2^) contrast with the uneven spatial distribution of anthropogenic impacts, and make the Western Siberian tundra a natural laboratory for studying the relative impact of environmental and anthropogenic drivers on tundra flora and vegetation across biological, temporal, and spatial scales.

**FIGURE 1:**
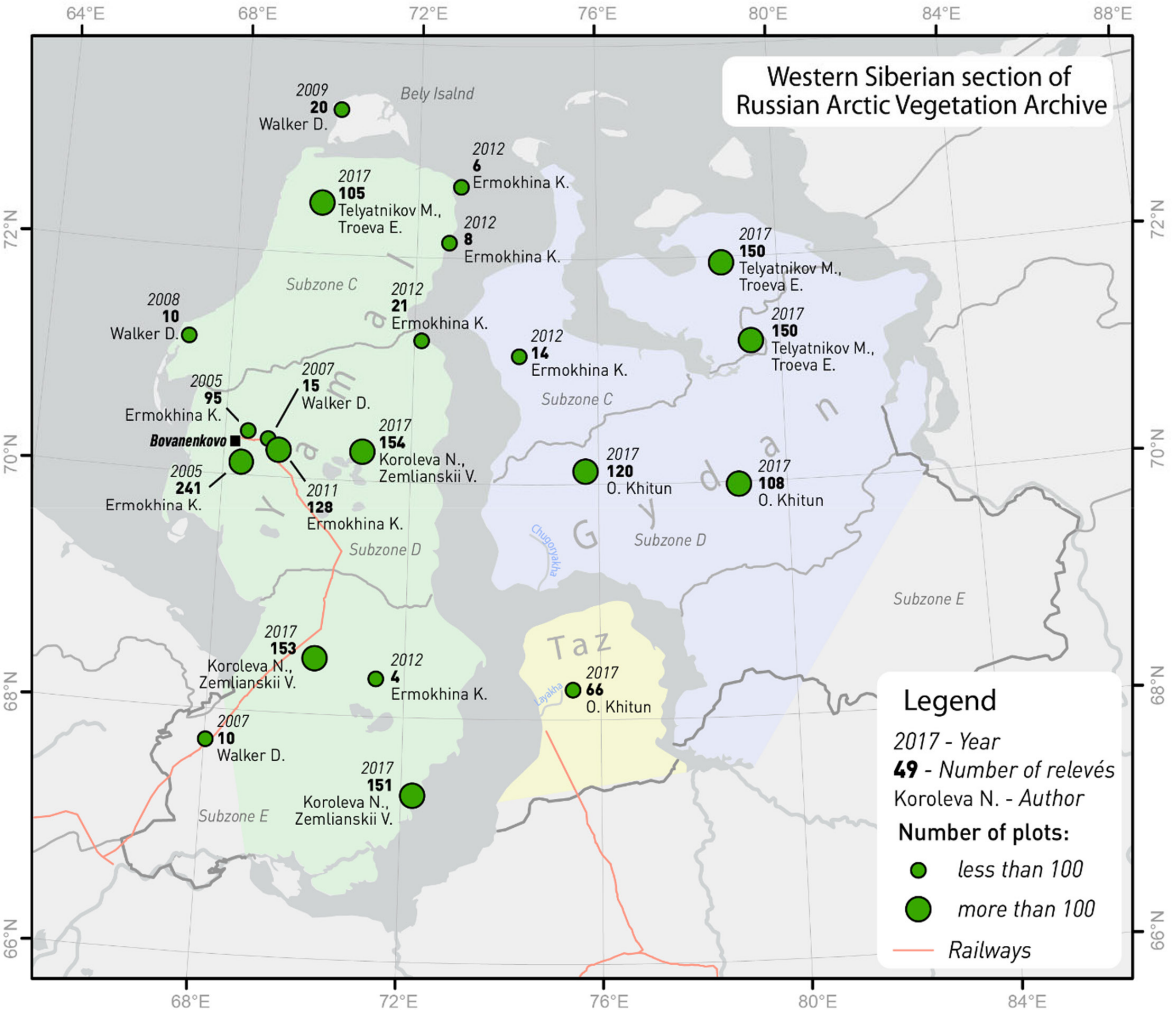
Western Siberian study area, including the location of the major study sites and respective number of geobotanical plots per site (= number of relevés). The Yamal peninsula is shaded in green, Taz in yellow, and Gydan in blue.

Most of the botanical research in the Western Siberian tundra was conducted at the site level, following the ‘local flora’ methodology (Khitun, 2002, 2003; Khitun et al., 2016; Khitun & Rebristaya, 1998; Rebristaya, 2013; Rebristaya et al., 1989; Rebristaya & Khitun, 1994, 1998). This methodology is based on a complete assessment of vascular plant species in an area of 100–300 km^2^. There are 42 local floras described across the Western Siberian tundra, but their distribution is uneven: about two thirds of the local floras were described on the Yamal peninsula, while other areas are poorly sampled. Local species pools vary widely: from 215 species in Layakha, west of Taz peninsula (Figure 1), subzone E (CAVM, 2003; Rebristaya et al., 1989), and 209 species in Chugoryakha, south‐west of Gydan (Figure 1), subzone E (CAVM, 2003; Rebristaya & Khitun, 1994), to 75 species on Bely Island, subzone B (CAVM, 2003; Rebristaya, 1995). Generally, regional species richness declines with latitude, but areas at the same latitude at Gydan have richer floras than at Yamal by 20–30 species (Khitun, 1998, 2016; Rebristaya, 2013). Although overall summer warmth has been identified as the main contributing factor to floristic richness gradients, other factors such as soil acidity, local topography, glaciation, and sea level history of the area are also considered important (Khitun, 1998, 2016; Rebristaya, 2013; Walker et al., 2005). The particular importance of Pleistocene sea level changes has been documented, although accurate quantification of its impact on the contemporary flora has remained challenging due to the lack of Pleistocene palynological data for the region (Rebristaya, 2013).

While earlier studies based on local floristic data provide important insight into regional vascular plant species richness, we still lack an understanding of which factors are structuring the species richness at the community level across the Western Siberian Arctic and how climate, topographic and anthropogenic factors combine to impact community species richness across large spatial extents. Large‐scale quantitative studies of community‐level species richness have not been carried out in Western Siberia, where existing studies rely either on traditional geobotanical methods or are limited to smaller areas (Forbes & Sumina, 1999; Khitun, 1998; Rebristaya, 2013). Here, based on a newly assembled, large geobotanical dataset (Zemlianskii et al., 2023), we aim to identify the main drivers and map the patterns of community‐level plant species richness, including vascular plants, mosses and lichens, in the Western Siberian tundra. We estimate the relative impact of different contemporary and historical environmental and anthropogenic factors on plot‐level community species richness using macroecological models (Table S3). We model and map the spatial distribution of mean plant species richness across the area and discuss these predictions in context of previous geobotanical studies. We hypothesize that (1) climate factors are more important in explaining patterns of community‐level species richness across vast Arctic plains than topographic factors, (2) paleoclimatic factors have higher explanatory power compared to the current climate, (3) anthropogenic factors are as important predictors as natural factors, (4) community‐level plant species richness in the area follows the latitudinal diversity gradient, and (5) current protected areas do not sufficiently well cover regions with high species richness.

## 2 METHODS

The objective of our research is to estimate the distribution of plant species richness at the community level across the Western Siberian tundra. To this end, we calibrated macroecological models, predicting mean plot‐level plant species richness as a function of environmental factors (Guisan et al., 2017; Guisan & Rahbek, 2011) from geobotanical plots sampled across the region. We also estimated the role of anthropogenic factors, using distance from infrastructure as a proxy for anthropogenic impact.

### 2.1 Study area

The Western Siberian tundra is located in the northern part of the Western Siberian plain and covers slightly more than 300,000 km^2^. The area has a low plant species richness at the regional level because of its landscape properties and geoclimatic history. The area belongs to the European‐West‐Siberian province (Yamal‐Gydan subprovince) of the Arctic floristic region (CAVM team, 2003; Yurtsev, 1994). In comparison with neighboring subprovinces, Yamal‐Gydan is characterized by almost complete absence of endemism, low vascular plant species richness (the lowest in continental Russia), and a lack of many montane species (Daniëls et al., 2013; Khitun, 1998; Rebristaya, 2013; Sekretareva, 1999; Yurtsev, 1994). In total, the province harbors about 450 species of vascular plants (Koroleva et al., 2011), 276 species of mosses (Chernyadyeva, 2001; Voronova & Diachenko, 2018) and 250 species of lichens (Magomedova et al., 2006). The flora of the area was shaped by Quaternary climate oscillations as well as marine transgressions and (to lesser extent) glaciations, which had an especially strong impact on Yamal (Rebristaya, 2013; Stewart et al., 2016). Landscape homogeneity, high soil acidity, and the absence of bedrock exposure also contribute to observed low species richness (Khitun, 1998; Rebristaya, 2013).

### 2.2 Geobotanical plots

To estimate community‐level species richness, we used geobotanical data from the Russian Arctic Vegetation Archive (Ermokhina et al., 2022; Zemlianskii et al., 2023). These data consist of 1483 Braun‐Blanquet plots established in homogenous vegetation collected during the 2005–2017 field campaigns in the Western Siberian tundra (Figure 1) (Zemlianskii et al., 2023). The data were collected following the standard international Arctic Vegetation Archive protocol (Walker et al., 2013, 2016, 2018) and include full species lists of vascular plants and, contrary to most other existing floristic studies of the area, also bryophytes and lichens (Elven et al., 2011; Raynolds et al., 2013). For the 12 major sites (100–150 km^2^ sub‐areas, with more than 60 plots sampled in each), we collected data representative for all vegetation types found in the area (at least 5 plots per community per major site). In addition, we used 10 minor sites with 4–21 plots per site. The plot size varied from 25 to 100 m^2^ depending on community characteristics (Matveeva, 1998). We divided plots into two classes, large plots (100 m^2^) and small plots (less than 100 m^2^), to test for the effect of plot size on species richness.

The plot‐level species richness, which we calculated as plot‐wise numbers of present species of vascular plants, mosses, and lichens (liverworts data were omitted because of uneven identification quality across the database), was used to build regression‐type macroecological models. The response variable of our models was species richness per community. To estimate latitudinal trends at the site‐level, we also inferred lichen, moss, vascular plants, and total species richness for each major site.

### 2.3 Predictor variables

For each geobotanical plot, we first extracted co‐located data from an initial set of 48 contemporary environmental predictors describing climate, topography, vegetation productivity, and anthropogenic impact (Table S1). Climatic predictors included wind speed from the Global Wind Atlas (Davis et al., 2023; https://globalwindatlas.info/), 19 bioclimatic variables (seasonal and annual statistics of temperature and precipitation) from CHELSA (Karger et al., 2017), mean ground temperature from ESA Global permafrost project (Obu et al., 2019), and annual statistics of climate moisture index, total cloud cover, potential evapotranspiration, site water balance, and growing degree days from CHELSA‐BIOCLIM+ (Brun et al., 2022). Topographic predictors included altitude, standard deviation of altitude, topographic position index, log‐transformed slope, and aspect, which were derived from the Arctic digital elevation model (Morin et al., 2016; Porter et al., 2018), and topographic wetness index (Marthews et al., 2015). Mean normalized difference vegetation index (NDVI) for the period July–August 2019–2020 as observed by MODIS (ORNL DAAC, 2018; https://modis.gsfc.nasa.gov/) was used as vegetation‐related predictor.

In addition to these contemporary environmental factors we tested the effect of five paleoclimatic variables (mean annual temperature, annual precipitation sum, paleo‐elevation, distance to land ice, and maximum (latest) year in the time‐series when the location was covered by land ice) since the Last Glacial Maximum period (221 time steps with 100‐year temporal resolution extending up to 22.000 years ago), originating from the CHELSA‐TraCE21k dataset (Karger et al., 2023) (Figures S4–S6). Information on these variables at the locations of our geobotanical plots was extracted using a publicly available R script (Assmann, 2023; https://github.com/jakobjassmann/cryo_db_v2). For paleoclimatic variables we (1) identified the timesteps with highest explained deviance for each predictor and (2) performed a selection of optimal timesteps comparing them with other predictors within the full set.

In addition to the environmental predictors mentioned above, we used distance to infrastructure as a proxy for anthropogenic impact, combining disturbance through industrial activities, and increased potential for species invasion into a single predictor. To this end, we downloaded all available data for roads, railroads, settlements, industrial sites, and airports from OpenStreet Map (https://www.openstreetmap.org) and converted them to points. Then, we calculated the distance between each standard grid raster cell and the closest infrastructure point using the nearest neighbor method.

We added distance to infrastructure as a predictor to the macroecological models to test its explained deviance. To assess if a possible effect on the model outcome is independent of environmental predictors, we generated a residual plots of the GAM model fitted with environmental predictors against distance to infrastructure predictor. Additionally, we tested the relationship between distance to infrastructure and the presence of those 413 species (out of the 840 species) with 10 or more occurrences individually. To do this, we fitted logistic regression models and looped through all 413 species, calculating *p*‐values and regression coefficients.

From the full set of predictors, we performed a selection for the final model calibration based on univariate predictive performance (see Table S1) and limited collinearity (absolute pairwise Pearson correlation coefficients <.7). The results of the selection were used in the final macroecological models.

Raster layers of all predictors were reprojected in QGIS (version 3.12, https://www.qgis.org/) to a standard grid in ESRI:102025 projection with 1000 m horizontal resolution. The resampling and predictor selection was conducted in R (version 4.1.2, R Core Team, 2021) using the packages raster and ecospat (Broennimann et al., 2014; Hijmans et al., 2015).

### 2.4 Fitting and validating macroecological models

We modeled species richness as a function of non‐anthropogenic predictors using four different model algorithms: random forest (RF, Breiman, 2001), gradient boosting machine (GBM, Friedman, 2001), generalized linear model (GLM, McCullagh & Nelder, 1983), and generalized additive model (GAM, Hastie & Tibshirani, 1990) (see Table 1). For RF, we fitted 500 regression trees, considering three predictors for each tree. For GBMs, we set the number of trees to 80, the minimum number of data points per leaf to 10, the learning rate to 0.1 and the error distribution to ‘poisson’. For GLM and GAM we assumed a Poisson error distribution and used the ‘log’ link function. For GLMs, we defined linear and quadratic terms for each predictor. For GAMs, we used smooth terms with four degrees of freedom. For GLM and GAM, we step‐wise optimized the Akaike information criterion by removing uninformative predictor terms from the model equation.

**TABLE 1:** Model performance statistics from five‐fold cross‐validation.

Model	Spearman correlation (.58 on average)	Mean absolute error (8.1 on average)	Root mean square error (10.2 on average)
GLM	.60	8.0	10.1
Random Forest	.57	8.2	10.1
GAM	.56	8.2	10.2
GBM	.59	8.0	10.5

Abbreviations: GAM, generalized additive model; GBM, gradient boosting machine; GLM, generalized linear model.

Macroecological models were fitted in the R environment (version 4.1.2) using the packages randomForest (Liaw & Wiener, 2002), gbm (Greenwell et al., 2020), and gam (Hastie, 2020).

We used five‐fold cross‐validation to estimate model performance. Agreement between observed and predicted species richness was assessed using Spearman correlation coefficients, root mean square error (RMSE), and mean absolute error (MAE).

### 2.5 Spatial projections

We ensembled the spatial projections of species richness of all fitted models (Table 1). Ensemble predictions were generated using the mean of modeled species richness of the four different models. In addition, we derived the model disagreement between models as the prediction span (i.e., maximum—minimum predicted species richness among models per pixel) and displayed our plot locations on the model disagreement map in order to assess the effect of sampling bias on prediction uncertainty (Figure 3). Finally, we intersected the obtained richness map with a shapefile of the borders of Arctic protected areas in our study region (Arctic Council, Conservation of Arctic Flora and Fauna Working Group, 2010; CAFF, 1997) using rgdal package (Bivand et al., 2021).

## 3 RESULTS

### 3.1 The role of contemporary environmental factors

Testing the predictive power based on univariate predictive performance of the initial set of 48 environmental variables shows that climate‐related factors are better predictors of mean community‐level plant species richness than factors related to topography or distance to infrastructure (Table 2, Table S1). Our results confirm that community‐level species richness in the Arctic is strongly linked to warmth, but the relationship varies depending which seasonal temperature statistics is considered. Lower mean January temperature is associated with higher species richness (found primarily on the more continental Gydan peninsula) although the relationships are non‐linear as the warmest temperature (found at the more oceanic cost of eastern Yamal) is also associated with higher species richness compared to colder temperatures of central Yamal and northernmost Bely island (Figure S1c). The trend is different for mean daily maximum air temperature of the warmest month where highest species richness is associated with high temperatures (Figure S1f). Moisture factors are also important: both the annual maximum and annual range of the climate moisture index, and maximum and minimum monthly potential evapotranspiration have relatively high adjusted explained deviance (Figure S1b, Table S1). Cloud area fraction and mean wind speed show moderate explained deviance (5% and 7%, respectively) (Table S1). High species richness is associated with relatively low wind speed and cloud fraction. Topographic relief factors are generally less important for community‐level species richness than climate variables. Plant species richness is positively correlated with slope (Figure S1e) and standard deviation of altitude (Table S1). The latter two are the only two topographic relief predictors with an explained deviance higher than 5%. Altitude, aspect, topographic wetness index, and roughness (topographic position index), on the other hand, have very low explained deviance (Table S1).

**TABLE 2:** Environmental variables used in the models.

No.	Predictors	Explained deviance (%)	Original spatial res. (m)	Source
1	Mean annual paleotemperature (12,100 years ago)	21	30 arcsec (<1000)	CHELSA‐TraCE21k dataset (Karger et al., 2023)
2	Climate moisture index (max)	14	30 arcsec (<1000)	CHELSA new (Brun et al., 2022)
3	Mean January temperature	13	1000	MODIS derived 2000–2019 (MOD11A2 MODIS/Terra Land Surface Temperature/Emissivity 8‐Day L3 Global 1 km SIN Grid V006 [Dataset])
4	Mean annual paleoprecipitation (17.200 years ago)	12	30 arcsec (<1000)	CHELSA‐TraCE21k dataset (Karger et al., 2023)
5	Mean daily maximum air temperature of the warmest month (BIO10_05)	11	30 arcsec (<1000)	CHELSA Bioclim (Karger et al., 2016)
6	Isothermality (BIO10_03)	10	30 arcsec (<1000)	CHELSA Bioclim (Karger et al., 2016)
7	(log transformed) slope	10	10	ArcticDEM based (Morin et al., 2016; Porter et al., 2018)
8	Distance to land ice (9300 years ago)	7	30 arcsec (<1000)	CHELSA‐TraCE21k dataset (Karger et al., 2023)

*Note*: The full list of evaluated variables is presented in Table S1.

### 3.2 The importance of paleoclimatic predictors

Paleoclimatic predictors show high explained deviance, partly even higher than any contemporary temperature predictor used, yet they are strongly correlated with contemporary climate predictors. The strongest paleoclimate predictor is temperature from 12.1 thousand years ago, which alone explains 21% of the deviance (1% higher than that of the actual mean annual air temperature—the strongest contemporary temperature‐related predictor) (Figure S2a). The four strongest paleo‐predictors are all temperatures from the Pleistocene–Holocene boundary period (11.2–12.7 thousand years ago) and have high explained deviance (≈20.9%), while temperatures of 17–22 thousand years ago have lowest explained deviance (12%–15%) (Figure S6). At the same time, the strongest paleoclimatic predictors (temperature, precipitation, distance to land ice) exhibit high correlation with current mean ground temperature (.95, .83, and .72, respectively, for a 12.1‐thousand‐year‐old time point) and generally also among each other. The data show no evidence of the presence of either land ice or sea water at the plot locations throughout the entire time period since the Last Glacial Maximum.

### 3.3 Anthropogenic impact

To estimate the anthropogenic impact on species richness, we analyzed the distance to infrastructure as predictor, which shows moderate explained deviance (11%). At the same time, additional GAM residual tests show limited independent impact of distance to infrastructure on total plant species richness (Figure S2). Testing the relationships between single species and distance to infrastructure shows that 159 species exhibit significant positive relationships (91 highly significant), while 92 show negative relationships (38 highly significant) with distance to infrastructure (Figure S3, Table S2). Based on the additional test results distance to infrastructure was excluded from final model projection map.

### 3.4 Selected predictors and model performance

The final set of eight environmental predictors used for model calibration included four contemporary climate predictors (annual maximum of climate moisture index, mean January temperature, mean daily maximum air temperature of the warmest month, and isothermality), three paleoclimatic predictors (mean annual temperature 12,100 years ago, mean annual precipitation 17,200 years ago, and the distance to land ice 9300 years ago), and one topographic predictor (log‐transformed slope) (Table 2). Plot size was omitted as a predictor during GAM and GLM stepwise variable reduction, so we consider the plot size effect as minor as long as the area of the plots lies within the range assessed here. Multivariate GBM and RF also show the same model performance with and without the use of plot size. A detailed list of all tested and selected predictors can be seen in Table S1.

Using the eight selected predictors, GAM, GLM, RF, and GBM show close performance statistics (Table 1). Our model predictions to the left‐out cross‐validation subsets showed a Spearman correlation of .58, an RMSE of 10.2 and MAE of 8.0. The best model was GLM with a Spearman correlation of .60 and a MAE of 8.0.

The ensemble of models shows low model disagreement (less than 5 species) in most parts of Gydan, Taz peninsula, and some areas of Northern and coastal Western and Eastern Yamal (Figure 3). We identified high model uncertainty (10–15 species) in Southern and Central Yamal, around Bovanenkovo in the West, and the South Tambey gas field at the eastern coast of Yamal.

### 3.5 Spatial patterns of community‐level plant species richness

**FIGURE 2:**
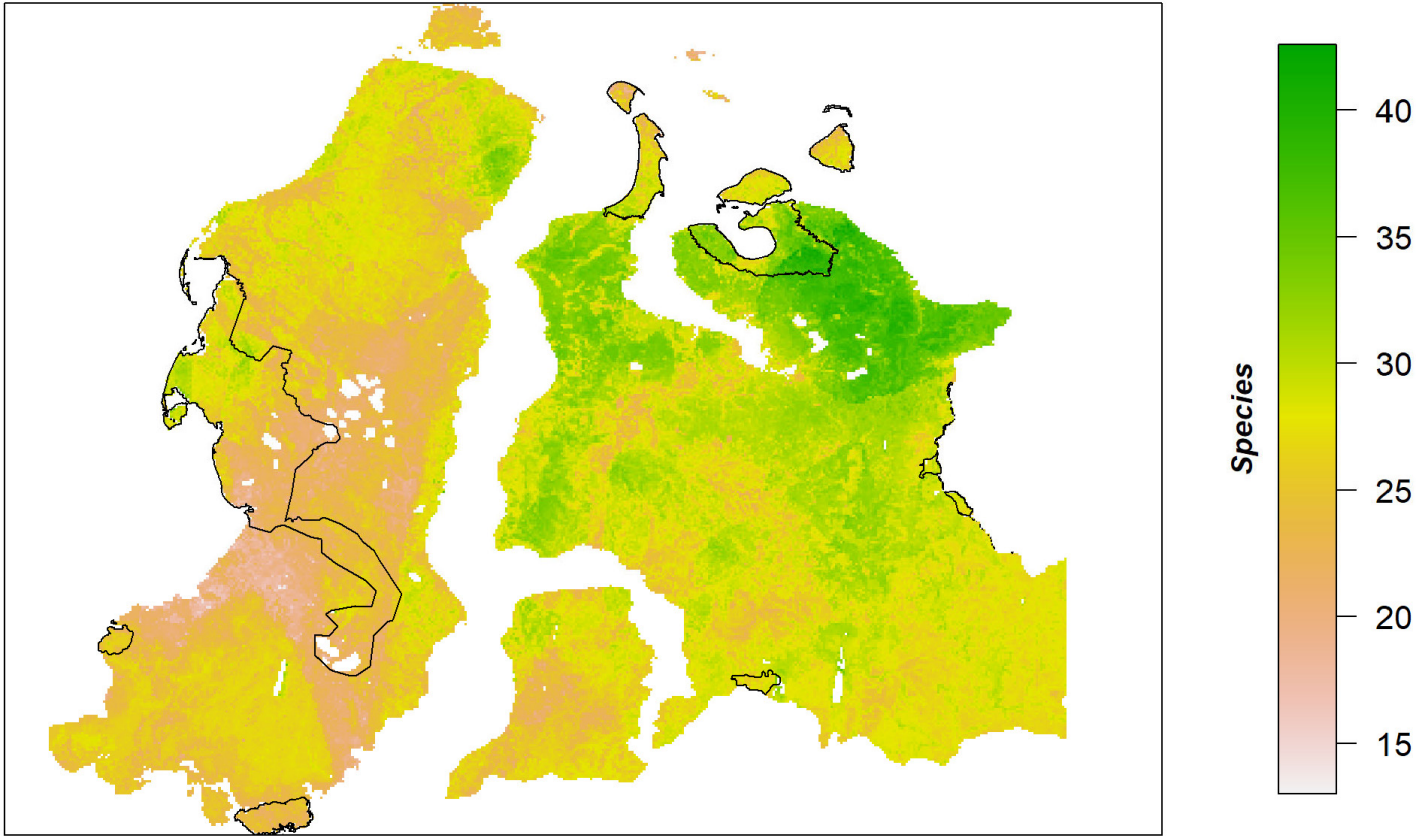
Mean plant species richness distribution in the Western Siberian tundra as predicted by a macroecological model ensemble based on a general additive (GAM), general linear (GLM), gradient boosting machine (GBM), and random forest (RF) model. Black borders show existing protected areas.

**FIGURE 3:**
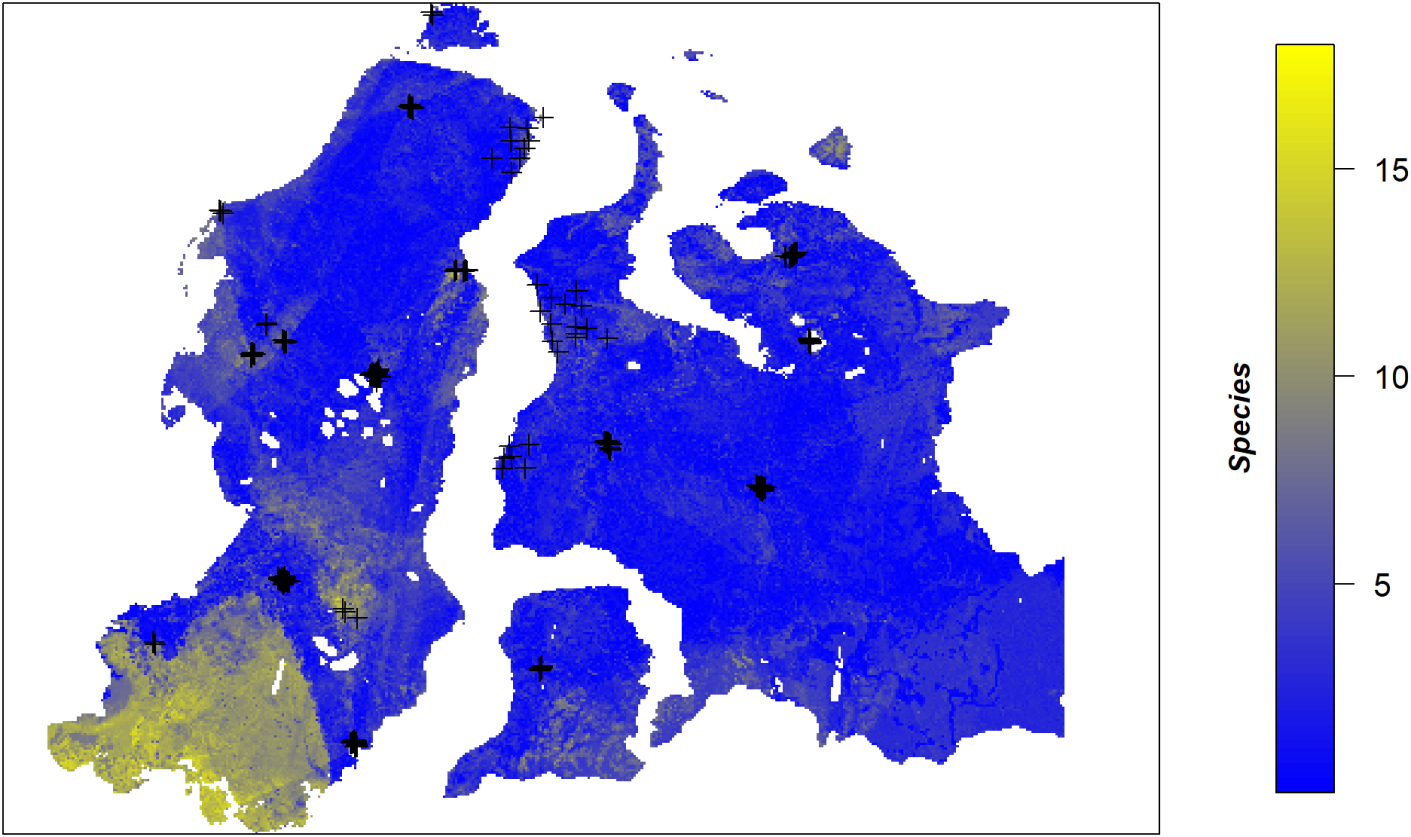
Model disagreement map indicating maximum difference in predicted species number between GAM, GLM, GBM, and Random forest. Black crosses indicate geobotanical plot locations.

Our model results show a highly heterogeneous distribution of community‐level plant species richness across the Western Siberian tundra (Figure 2). Mean species richness of the model ensemble map varies from 15 species on Eastern Yamal, Bovanenkovo railroad area (Figure 3), to more than 40 in the Gydan National Park area. The Yamal peninsula shows generally lower species richness than Gydan. Furthermore, longitudinal differences between Yamal and Gydan are generally higher than latitudinal differences within both peninsulas. Protected areas (except Gydan National Park) generally cover areas with low species richness. Importantly, the main part of the species‐rich area in Northern Gydan remains unprotected as well as smaller species‐rich areas in Northern and Eastern Yamal.

It is widely recognized that landscape‐level or regional plant species richness in the Arctic tundra is strongly dependent on summer warmth and hence declines with latitude. At the community level, we found an opposing trend: median species richness of lichens, mosses, and vascular plants increases with latitude (reduced warmth) (Figure 2). Based on the univariate predictive performance, latitude is a relatively strong predictor of community‐level plant species richness across the area with 15% of deviance explained (Table S1). Given its high correlation with other, more direct predictors, latitude was not used in the final model. However, we clearly see a temperature‐richness effect that is opposed to the expected decline of richness with latitude and associated decrease in temperature.

## 4 DISCUSSION

Our models reveal a highly heterogeneous spatial distribution of community‐level plant species richness across the Western Siberian Arctic. In the study, we tested five hypotheses. As we expected in H.1 climate factors such as temperature and precipitation play key roles in shaping community species richness while topography plays a secondary role (Table 2). Paleoclimatic factors are shown to be stronger predictors compared to similar contemporary climatic factors, while being strongly correlated with the latter (H.2). While revealing relatively high explained deviance, additional statistical tests showed that the effect of distance to infrastructure on plant species richness is difficult to interpret, contrary to H.3. Contrary to the pattern common in the Arctic at regional scale, we do not find a latitudinal decrease in community‐level species richness from South to North, but rather a consistent increase from South‐West to North‐East (H.4, Figure 2). Finally, in accordance with H.5, our analysis suggests that the most species‐rich areas remain largely unprotected (Figure 2).

Our results unveil the complex interplay of factors driving community species richness in the Western Siberian tundra. Among contemporary predictors, climate is showing the strongest influence on plant species richness patterns in Western Siberian Arctic. Between climate predictors, temperature‐related factors such as mean annual and mean ground temperature, growing degree‐days and mean January temperature best explained plant species richness which is in line with previous Arctic studies (CAVM, 2003). Moisture factors are also important—a high annual climate moisture index range is associated with high species richness, presumably because continental parts of the region have higher habitat diversity than oceanic ones. Conversely, areas with high cloud area fraction are associated with low species richness as sunlight is an important limiting factor for plant life in the tundra (Chapin, 1987). Topography‐related factors and wind speed are generally weaker predictors of plant species richness than climatic factors. The impact of altitude itself on species richness is low due to the generally flat terrain in our study area and hence low variability in altitude. However, we have demonstrated that terrain roughness, as indicated by factors such as slope and the standard deviation of altitude, is associated with high species richness. This finding aligns with previous research, as some of the species‐rich communities in the area, such as tundra meadows, are often found on steep slopes (Rebristaya, 2013; Telyatnikov, 2005). The role of wind speed is less conclusive because areas with the lowest wind speed have few geobotanical plots. However, the areas with highest wind speed exhibit lower species richness, which is in good agreement with the known negative impact of wind erosion (deflation) on Yamal tundra ecosystems (Ektova, 2008; Ermokhina & Myalo, 2012a, 2012b).

The testing of historical climate predictors indicates that paleoclimate had the strongest impact on plant species richness distribution. Notably, some paleoclimate predictors such as temperature and precipitation exhibited higher explained deviance than their contemporary counterparts, which indicates a legacy effect of past climate on the contemporary community‐level richness patterns (Stewart et al., 2016). According to the CHELSA‐TraCE21k dataset, our study area was not affected by glaciation or sea level change over the past 21,000 years, which differs from previous research on the region that indicated some sea transgressions during the Boreal age of the Holocene (9200–8200 years ago), although not as pronounced as those in the Pleistocene (Rebristaya, 2013). It is challenging to separate the influence of contemporary from historical climate, as demonstrated at the example of Gydan, where the high species richness is mostly attributed to its historical development (Khitun, 1998).

We found no conclusive evidence that distance to infrastructure affects species richness in Western Siberia. Despite strong evidence of impact of anthropogenic activities on the vegetation of the region (Ektova & Morozova, 2015; Ermokhina et al., 2023; Forbes, 2013; Golovatin et al., 2010; Golovnev et al., 2016; Veselkin et al., 2021), a sensitivity analysis suggests that most of the impact of the distance to infrastructure predictor is attributable to other predictors (Figure S2). At the same time, indirect indicators such as relatively high explained deviance of the distance (11%) show that there might be a potential relationship that cannot be confidently detected with the data available. The spatial distribution of some species (such as *Deschampsia brevifolia* R. Br. or *Poa alpina v. vivipara* L.) which were found primarily at closer distance to infrastructure (i.e., with significant negative correlations) in our analysis are indeed classified as apophytes (Sekretareva, 1999). In other cases, especially for many species showing strong negative relationships with distance to infrastructure (f.e. *Dactylina ramulosa* Hook. Tuck.), sampling bias (due to a sampling gap on intermediate to long distances) may have played a role. Our model results indicate that a better designed spatial sampling is needed to investigate the direct and indirect impact of human activities, such as industrial expansion and related herding density change, on spatial patterns plant species richness.

Our results suggest increasing mean community‐level species richness from South‐West to North‐East which is the opposite of the common view of a distinct negative latitudinal richness gradient in the Arctic (Daniëls et al., 2000, 2013; Schultz, 2005; Walker et al., 2005), but in agreement with some earlier site‐level studies in Western Siberia (Khitun, 1998; Rebristaya, 2013). We consider the following four main reasons for this consistent but somewhat unexpected increase in plot‐level species richness from South‐West to North‐East in this vast Arctic plain:

1. Climatic gradients (temperature, precipitation, seasonality) do not follow the typical South–North direction of the northern hemisphere in our study region, which might be linked to increasing continentality from West to East, supporting a wider range of plant species. We show that contemporary climate has a strong impact on community‐level species richness. Specifically, lower mean temperature in January and higher mean daily maximum air temperature during the warmest month are associated with higher species richness (see Figure S1c,f). The combined impact of these two temperature factors is an indicator of continentality and has a positive influence on species richness.
2. Topographic variability: variations in elevation, slope, and aspect can create diverse microclimates and soil conditions, supporting a broader spectrum of plant species adapted to specific ecological niches within the landscape. The Gydan peninsula in the East of our study area shows larger variation in topography compared to the rather flat Yamal and Taz peninsulas. Topography has been shown to play an important while clearly secondary role as hilly areas with steeper slopes in coastal and northern Gydan show a higher species richness, as confirmed by our models (Figure 2, Figure S1e).
3. Anthropogenic factors: The patchy but rather low predicted species richness in the southern and western parts of the study area (especially in southern and central Yamal) might also partly result from a combination of intense reindeer herding and land use change related to gas extraction. Although we found some indication of anthropogenic influence, we lack conclusive evidence to demonstrate that distance to infrastructure influences regional species richness on a broad spatial scale.
4. Historical factors: past geological and ecological events, such as sea level change, glaciation patterns, timing of post‐glacial colonization, and post‐glaciation climate change, can leave lasting legacies on vegetation patterns. The strong relationship between the contemporary status of vegetation and the history and (paleo)geography of the region was hypothesized to play a key role in earlier studies (Khitun, 1998; Rebristaya, 2013). Northern Gydan, which contained refugia during the last ice age (Khitun, 1998), has a higher richness than the Yamal peninsula. The latter was completely covered by water during the middle Pleistocene and mostly during the late Pleistocene transgressions, while Gydan kept the terrestrial connections with the relatively rich Taymyr and Central Siberian floras (Khitun, 1998; Rebristaya, 2013). The transgressions from the middle to late Pleistocene fall outside the temporal extent of the paleoclimate dataset used in our study. However, we show that the current spatial distribution of species richness is well explained by late Pleistocene and Holocene paleoclimate, indicating a lasting impact of historical factors on species richness in the Western Siberian Arctic.

Several non‐quantified factors might also influence the species richness pattern. The described inverse trend in mean community‐level species richness over the West Siberian Arctic could be strengthened by high reindeer grazing densities in Southern Yamal, documented by previous studies (Veselkin et al., 2021). The north‐east of Gydan is also characterized by soils generally less acidic than Yamal, Taz and south‐west of Gydan permitting several arctic‐alpine species to migrate from the east and contribute to the high species richness of the area (CAVM, 2003; Khitun, 1998). Unfortunately, there is a lack of comprehensive, high‐resolution and spatially well‐sampled data on soil pH and reindeer density, making it challenging to incorporate them in our models.

We conclude that plant species richness across the Western Siberian tundra is shaped by a combination of environmental and anthropogenic factors, whereby the influence of (paleo‐) climate factors is strongest. Our study shows that the capacity of nature reserves to protect plant species in our study area is limited because of the insufficient spatial coverage of areas with highest species richness. In addition to this finding, additional factors, such as the low spatial connectivity between protected areas, their focus on animal protection, and their often weak protection status are caveats for conservation efforts in this area (Barry et al., 2017; Kalyakin et al., 2000). Plant diversity protection requires a complex social‐ecological approach that is up to be developed. More targeted evaluation of the impact of industrial development on plant species richness and active participation of Nenets people should be part of the approach towards an effective action plan to protect plant species and their ecosystem functions in the Western Siberian Arctic.

## AUTHOR CONTRIBUTIONS

**Vitalii Zemlianskii:** Conceptualization (equal); data curation (lead); formal analysis (lead); funding acquisition (equal); methodology (equal); validation (equal); visualization (lead); writing – original draft (lead); writing – review and editing (equal). **Philip Brun:** Formal analysis (equal); methodology (equal); validation (equal); visualization (equal); writing – original draft (supporting); writing – review and editing (equal). **Niklaus E. Zimmermann:** Conceptualization (equal); formal analysis (supporting); methodology (equal); writing – original draft (supporting); writing – review and editing (equal). **Ksenia Ermokhina:** Conceptualization (equal); data curation (equal); formal analysis (supporting); methodology (supporting); validation (supporting); writing – original draft (supporting); writing – review and editing (equal). **Olga Khitun:** Data curation (equal); methodology (supporting); validation (equal); writing – original draft (supporting); writing – review and editing (supporting). **Natalia Koroleva:** Data curation (equal); validation (supporting); writing – original draft (supporting). **Gabriela Schaepman‐Strub:** Conceptualization (equal); data curation (supporting); funding acquisition (lead); methodology (equal); supervision (lead); writing – original draft (equal); writing – review and editing (equal).

## FUNDING INFORMATION

This work was supported by a Swiss federal scholarship (2019.0075).

## CONFLICT OF INTEREST STATEMENT

We declare no conflict of interest.

## Supporting information


FiguresS1–S6 and Table S1–S3.

## Data Availability

Data available from the Dryad Digital Repository (https://datadryad.org/stash/share/bFWEuics4IXhXfj2xvo4or1sUYa‐WriskoaRUuoVdeU) (Zemlianskii et al., 2023).
