# Supplementary material for: Current and past climate co‐shape community‐level plant species richness in the Western Siberian Arctic
Source: Ecol Evol. 2024 Mar 17;14(3):e11140. doi: 10.1002/ece3.11140 (PMC10944673; doi:10.1002/ece3.11140)
Supplement: Supplementary file 1 — FiguresS1–S6 and Table S1–S3. [file ECE3-14-e11140-s001.zip › Table S3.pdf]

Appendix Table 3: Current and past climate co-shape community-level plant species richness in the Western Siberian Arctic paper ODMAP protocol.

ODMAP element	Contents
**OVERVIEW**	
Authorship	• Authors: V. Zemlianskii, P. Brun, N.E. Zimmermann, K. Ermokhina, O. Khitun, N. Koroleva, G. Schaepman-Strub<br>• Contact email: vitalii.zemlianskii@ieu.uzh.ch<br>• Title: Current and past climate co-shape community-level plant species richness in the Western Siberian Arctic<br>• DOI: 10.1002/ece3.11140
Model Objective	• Objective: Mapping/Explanation<br>• Target output: predicted community-level species richness
Taxon	Vascular plants, mosses and lichens
Location	**Western Siberian tundra**, Russia
Scale of analysis	• Spatial extent (Lon/Lat): Longitude 66.8233 - 83.232754 E, Latitude 73.495569 N - 66.479605 N<br>• Spatial resolution: 1 km<br>• Temporal resolution and extent: resolution none; extent of field sampling 2005-2018<br>• Type of extent boundary: floristic (boundary of Yamal-Gydan floristic province (CAVM, 2003))
Biodiversity data overview	• Observation type: Community plots<br>• Response/Data type: species numbers
Type of predictors	• Climatic (including paleoclimate), topographic, anthropogenic
Conceptual model / Hypothesis	Species richness is co-shaped by natural (climate, topography, etc.) and anthropogenic factors
Assumptions	We assumed that (a) relevant ecological drivers (or proxies) of species richness are included, (b) detectability does not change across habitats, (c) sampling is adequate and representative(and any biases are accounted for/corrected), distance to infrastructure is an effective proxy to measure anthropogenic impact
SDM algorithms	• **Model algorithms**: We built macroecological models using GLMs, GAMs, GBMs and Random Forests algorithms. We use one ensemble method (the mean probability of occurrence from three best performing modelling algorithms).<br>• **Model tuning:** For GLMs and GAMs, we step-wise optimized the Akaike information criterion by removing uninformative terms from the model equation.<br>• **Model averaging/ensemble:** GAMs, GBMs, Random Forest were combined in an ensemble
Model workflow	We used General Linear Models, General Additive Models, Random Forests, and Gradient boosting to predict species richness as a response variable of environmental predictors selected based on univariate predictive performance, limited collinearity (absolute pairwise Pearson correlation coefficients <0.7), and ecological relevance. We estimated the

	role of anthropogenic factors using distance from infrastructure derived from Open Street Maps as a proxy for human influence.<br>Predictive model performance was assessed using a 5-fold cross-validation
Software	• **Software**: R (version 4.1.2, R Core Team, 2021), QGIS (version 3.12, https://www.qgis.org/)<br>○ **R-Packages used**: ecospat (Broennimann et al., 2014), gam (Hastie, 2020), gbm (Greenwell et al., 2020), randomForest (Liaw and Wiener, 2002), raster (Hijmans et al., 2015), and rgdal (Bivand et al. 2021).<br>• **Data availability:** https://datadryad.org/stash/share/bFWEuics4IXhXfj2xvo4or1sUYa-WriskoaRUuoVdeU<br>• **Code availability**: https://datadryad.org/stash/share/bFWEuics4IXhXfj2xvo4or1sUYa-WriskoaRUuoVdeU
**DATA**	
Biodiversity data	• **Taxonomic reference system**: We used Pan-Arctic species list (PASL) (Raynolds et al., 2013) as taxonomic reference<br>• **Ecological level**: community-level<br>• **Biodiversity data source**: We used Russian Vegetation Archive data (Ermokhina et al., 2022; Zemlianskii et al., 2023) for identifying community-level species richness<br>• **Sampling design:** Data was collected using standard Braun-Blanquet method according to Arctic Vegetation Archive protocol (Walker et al., 2013, 2016, 2018). Sample size varied from 16 to 100 m according to AVA protocol for tundra communities. Plots were classified the plots to small (less than 100m2) and large (100m2) to correct for the potential effect of plot size on species richness.<br>• **Sample size:** 1438 plots
Data partitioning	5-fold cross-validation
Predictor variables	• Predictor variables:<br>○ Climatic: 19 bioclimatic variables (seasonal and annual statistics of temperature and precipitation), mean ground temperature, annual statistics of climate moisture index, total cloud cover, potential evapotranspiration, site water balance, and growing degree days, mean wind speed<br>○ Paleoclimatic: mean annual temperature, annual precipitation sum, paleo-elevation, distance to land ice, maximum (latest) year in time-series where the location was covered by land ice.<br>○ Topography: altitude (incl. standard deviation of altitude), slope, aspect, topographic position index, terrain wetness index and solar radiation<br>○ Vegetation: Mean normalized difference vegetation index (NDVI) was tested but omitted during variable selection

<table>
<tr><td></td><td>Anthropogenic impact: Distance to infrastructure used as a proxy for anthropogenic impact, combining industrial activities and the resulting increase of reindeer pressure into one single predictor
Data sources:
Bioclimatic variables: CHELSA (Karger et al., 2017), and CHELSA-BIOCLIM+ (Brun et al., 2022).
Mean ground temperature (2000-2016): ESA Global permafrost project (Obu, et al., 2019)
Paleoclimate: CHELSA-TraCE21k dataset (Karger et al., 2021)
Terrain wetness index (Marthews et al., 2015)
Mean wind speed: Global Wind Atlas (https://globalwindatlas.info/en)
Topography: ArcticDEM based (Morin et al., 2016; Porter et al., 2018)
Vegetation: NDVI for the period July-August 2019-2020 as observed by MODIS (https://modis.gsfc.nasa.gov/)
Human impact: derived from Open Street Maps (https://www.openstreetmap.org/)

Data processing: slope, aspect and solar radiation as well as distance to infrastructure were calculated in QGIS (version 3.12, https://www.qgis.org/en/site/)
</td></tr>
<tr><td colspan="2">MODEL</td></tr>
<tr><td>Variable pre-selection</td><td>
The selection was based on univariate predictive performance (>5% explained deviance), limited collinearity (absolute pairwise Pearson correlation coefficients <0.7), and ecological relevance.
The final 8 variables mean annual paleotemperature (12.100 years ago), potential evapotranspiration (max), mean january temperature, mean annual paleoprecipitation (17.200 years ago), (log transformed) slope, mean daily maximum air temperature of the warmest month, isothermality, distance to land ice (9300 years ago).
</td></tr>
<tr><td>Multicollinearity</td><td>
We conducted Spearman’s rank correlations between all pairs of variables and dropped three variables that were highly correlated with others (Spearman’s $|\rho| < 0.7$) to reduce the risk of overfitting during model calibration.
</td></tr>
<tr><td>Model settings</td><td>
For GLMs, we defined a linear and a quadratic term for each predictor.
For GAMs, we used smooth terms with four degrees of freedom.
For GBMs, we set the number of trees to 80, the minimum number of data points per leaf to 10, learning rate equals to 0.1 and the distribution equals ‘poisson’
For Random Forests we fitted 500 regression trees, considering three predictors for each tree
</td></tr>
<tr><td>Model estimates</td><td>We used Spearman correlation and mean absolute error to estimate model performance.</td></tr>
</table>

Model averaging/ ensembles	We calculated the mean species richness from all models as consensus method for combining the output of different single-models.
Non-independent analyses	
**ASSESSMENT**	
Performance statistics	Performance statistics estimated on validation data (from data partitioning). Agreement between observed and predicted species richness was assessed using Spearman correlation coefficients and mean absolute error (MAE).
Plausibility checks	Maps of modelled predictions were checked by experts
**PREDICTION**	
Prediction output	**Prediction unit:** species numbers
Uncertainty quantification	We calculated model disagreement as the range between maximum and minimum predicted species richness in each pixel as measure of uncertainty.

References

Bivand, R., Keitt, T., & Rowlingson, B. (2021). rgdal: Bindings for the 'Geospatial'Data Abstraction Library. https://cran.r-project.org/src/contrib/Archive/rgdal/

Broennimann, O., Di Cola, V., Petitpierre, B., Breiner, F., Scherrer, D., Manuela, D., Randin, C., Engler, R., Hordijk, W., Mod, H., & Pottier, J. (2014). Package 'ecospat'. https://cran.r-project.org/web/packages/ecospat/index.html

Brun, P., Zimmermann, N.E., Hari, C., Pellissier, L., & Karger, D.N. (2022). Global climate-related predictors at kilometre resolution for the past and future, Earth Syst. Sci. Data Discuss. [preprint], https://doi.org/10.5194/essd-2022-212, in review.

CAVM team (2003). Circumpolar Arctic vegetation map." Conservation of Arctic Flora and 387 Fauna (CAFF) Map No 1.

Greenwell, B., Boehmke, B., Cunningham, J., & GBM Developers (2020). gbm: Generalized Boosted Regression Models. R package version 2.1.8.

Hastie, T. (2020). gam: Generalized Additive Models. R package version 1.20.

Hijmans, R. J., Van Etten, J., Cheng, J., Mattiuzzi, M., Sumner, M., Greenberg, J. A., ... & Hijmans, M. R. J. (2015). Package 'raster'. R package, 734, 473. ftp://h64-50-233-100.mdsnwi.tisp.static.tds.net/pub/cran/web/packages/raster/

Liaw, A. & Wiener, M. (2002). Classification and Regression by Random Forest. R News, 2, 18-22.

Karger, D.N., Conrad, O., Böhner, J., Kawohl, T., Kreft, H., Soria-Auza, R.W., Zimmermann, N.E., Linder, H.P., & Kessler, M. (2017). Climatologies at high resolution for the earth's land surface areas. Scientific data, 4(1), 1–20. https://doi.org/10.1038/sdata.2017.122

Karger, D. N., Nobis, M. P., Normand, S., Graham, C. H., & Zimmermann, N. E. (2021): CHELSA-TraCE21k v1. 0. Downscaled transient temperature and precipitation data since the last glacial maximum. Climate of the Past Discussions, 1-27.

Marthews, T. R., Dadson, S. J., Lehner, B., Abele, S., & Gedney, N. (2015). High-resolution global topographic index values. NERC Environmental Information Data Centre. https://doi.org/10.5285/6b0c4358-2bf3-4924-aa8f-793d468b92be

Morin, P., Porter, C., Cloutier, M., Howat, I., Noh, M.J., Willis, M., Bates, B., Willamson, C. & Peterman, K. (2016). ArcticDEM: a publically available, high resolution elevation model of the Arctic. Egu general assembly conference abstracts.

Obu, J., Westermann, S., Bartsch, A., Berdnikov, N., Christiansen, H.H., Dashtseren, A., Delaloye, R., Elberling, B., Etzelmüller, B., Kholodov, A., & Khomutov, A. (2019). Northern Hemisphere permafrost map based on TTOP modelling for 2000–2016 at 1 km2 scale. Earth-Science Reviews, 193, 299-316. https://doi.org/10.1016/j.earscirev.2019.04.023

Porter, C., Morin, P., Howat, I., Noh, M.J., Bates, B., Peterman, K., Keesey, S., Schlenk, M., Gardiner, J., Tomko, K., & Willis, M. (2018). ArcticDEM, V1, Harvard Dataverse. https://doi.org/10.7910/DVN/OHHUKH

Raynolds, M.K., Breen, A.L., Walker, D.A., Elven, R., Belland, R., Konstantinova, N., Kristinsson, H., & Hennekens, S. (2013). The Pan-Arctic Species List (PASL). Arctic Vegetation Archive (AVA) Workshop.

R Core Team (2021). Version 4.1. 2. R: A Language and Environment for Statistical Computing. R Foundation for Statistical Computing. Vienna: R Foundation for Statistical Computing.

Walker, D.A., Breen, A.L., Raynolds, M.K., & Walker, M.D. (2013). Arctic Vegetation Archive (AVA) Workshop.

Walker, D.A., Daniëls, F.J., Matveyeva, N.V., Šibík, J., Walker, M.D., Breen, A.L., Druckenmiller, L.A., Raynolds, M.K., Bültmann, H., Hennekens, S., & Buchhorn, M. (2018). Circumpolar arctic vegetation classification. Phytocoenologia, 48(2), 181–201. https://doi.org/10.1127/phyto/2017/0192

Walker, D.A., Daniëls, F.J.A., Alsos, I., Bhatt, U.S., Breen, A.L., Buchhorn, M., Bültmann, H., Druckenmiller, L.A., Edwards, M.E., Ehrich, D., & Epstein, H.E. (2016). Circumpolar Arctic vegetation: a hierarchic review and roadmap toward an internationally consistent approach to survey, archive and classify tundra plot data. Environmental Research Letters, 11(5), 055005. https://doi.org/10.1088/1748-9326/11/5/055005

Zemlianskii, V. , Ermokhina, K., Schaepman-Strub, G., Matveyeva, N., Troeva, E., Lavrinenko, I., Telyatnikov, M., Pospelov, I., Koroleva, N., Leonova, N., Khitun, O., Walker, D., Breen, A., Kadetov, N., Lavrinenko, O., Ivleva, T., Kholod, S., Petrzhik, N., Kurysheva, M., Gunin, Y., Lapina, A., Korolev, D., Kudr, E., & Plekhanova, E.. Russian Arctic Vegetation Archive – a new database of plant community composition and environmental conditions. Global Ecology and Biogeography. 2023. https://doi.org/10.1111/geb.13724

Zurell D., Franklin J., König C., Bouchet P.J., Serra-Diaz J.M., Dormann C.F., Elith J., Fandos Guzman G., Feng X., Guillera-Arroita G., Guisan A., Leitão P.J., Lahoz-Monfort J.J., Park D.S., Peterson A.T., Rapacciuolo G., Schmatz D.R., Schröder B., Thuiller W., Yates K.L., Zimmermann N.E., Merow C. (2020). A standard protocol for describing species distribution models. Ecography 43, 1261-1277. DOI: 10.1111/ecog.04960

Ermokhina K., Zemlianskii V., Kurysheva M., & Korolev D. Russian Arctic Vegetation Archive website. Retrieved June 30, 2022, from https://avarus.space/

Global Wind Atlas. Retrieved June 30, 2022, from https://globalwindatlas.info/en

Open Street map. Retrieved June 30, 2022, from https://www.openstreetmap.org/

ORNL DAAC 2018. MODIS and VIIRS Land Products Global Subsetting and Visualization Tool. ORNL DAAC, Oak Ridge, Tennessee, USA. Retrieved Semtember 23, 2021 https://modis.gsfc.nasa.gov/

QGIS Development Team (2022). QGIS Geographic Information System. Open Source Geospatial Foundation Project. Version 3.12. Retrieved June 30, 2022, from http://qgis.osgeo.org/
