# Supplementary material for: Current and past climate co‐shape community‐level plant species richness in the Western Siberian Arctic
Source: Ecol Evol. 2024 Mar 17;14(3):e11140. doi: 10.1002/ece3.11140 (PMC10944673; doi:10.1002/ece3.11140)
Supplement: Supplementary file 1 — FiguresS1–S6 and Table S1–S3. [file ECE3-14-e11140-s001.zip › Tables S1-S2.docx]

1. **Supplementary Material & Appendices**

| **a.**  **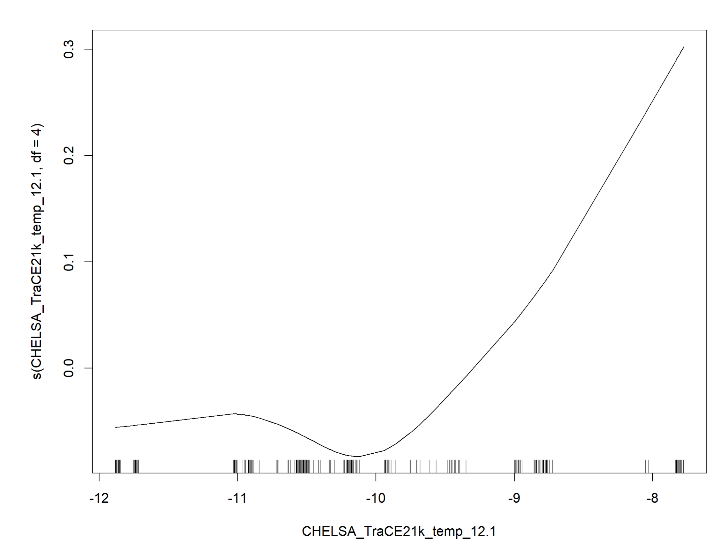** | **b. 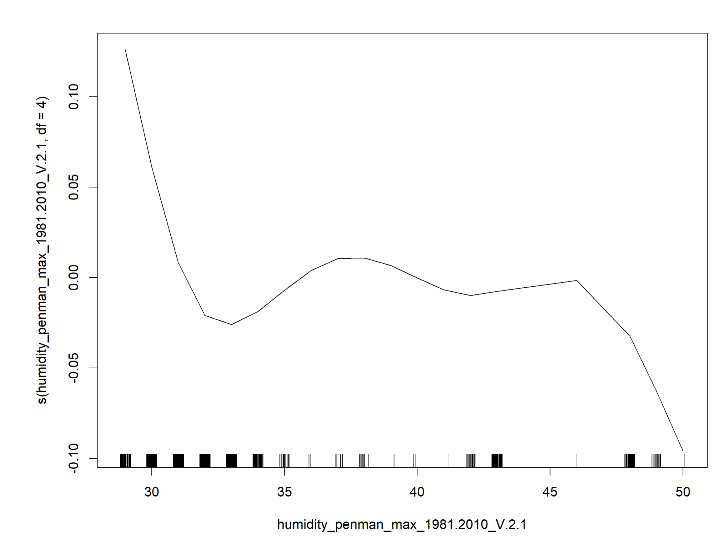** |
| --- | --- |
| **c. 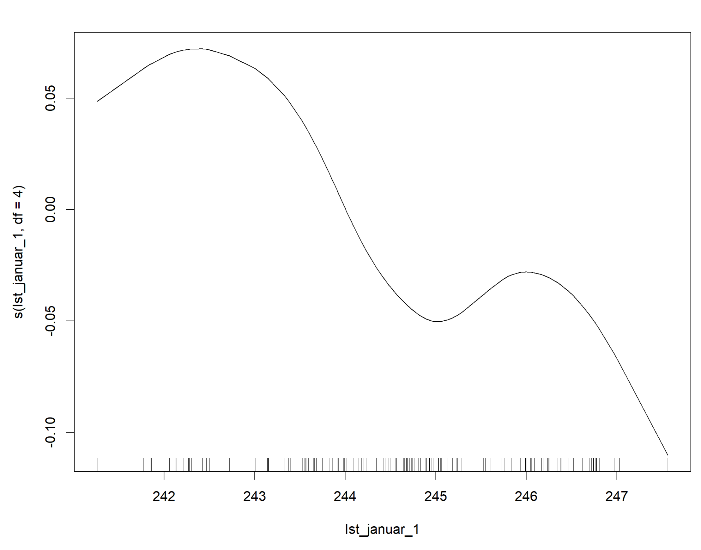** | **d. 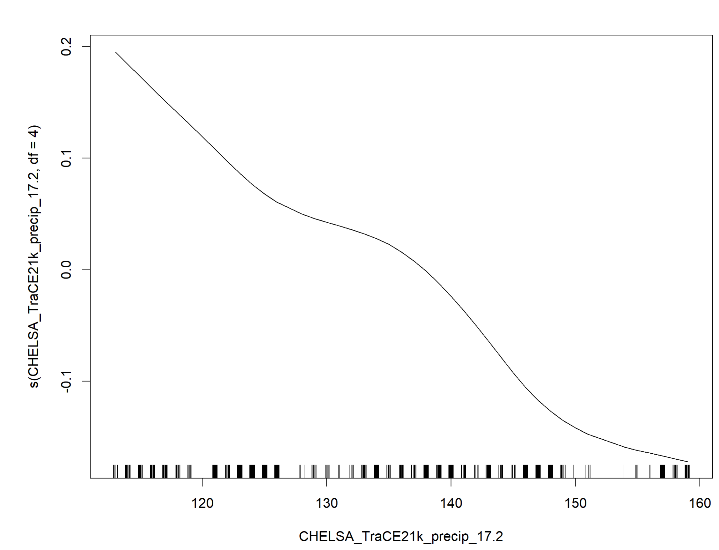** |
| **e.**  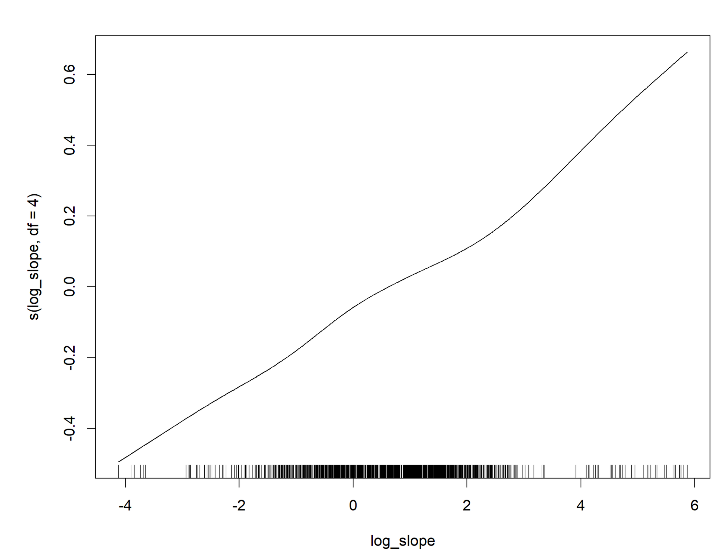 | **f.**  **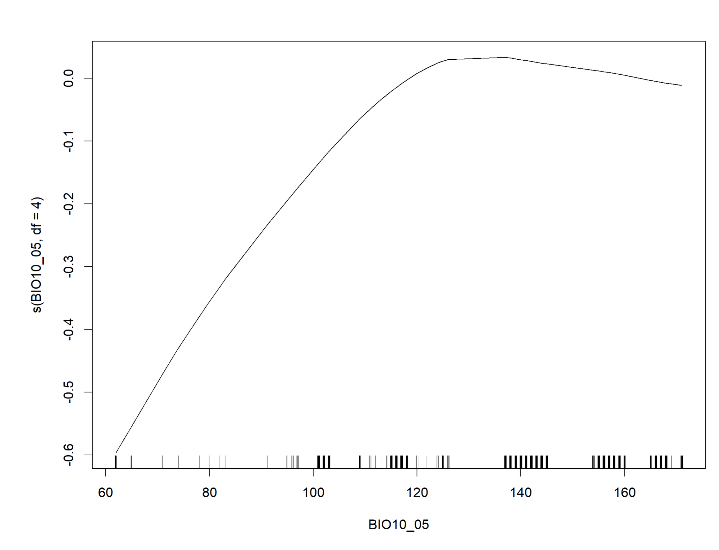** |
| **g. 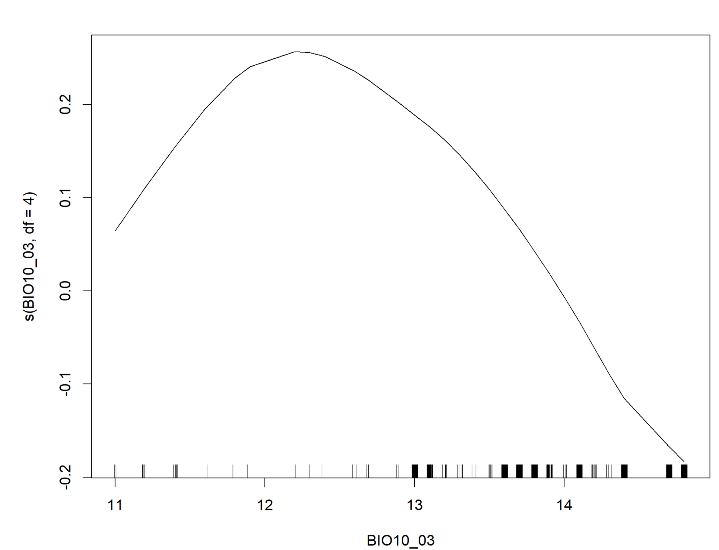** | **h.**  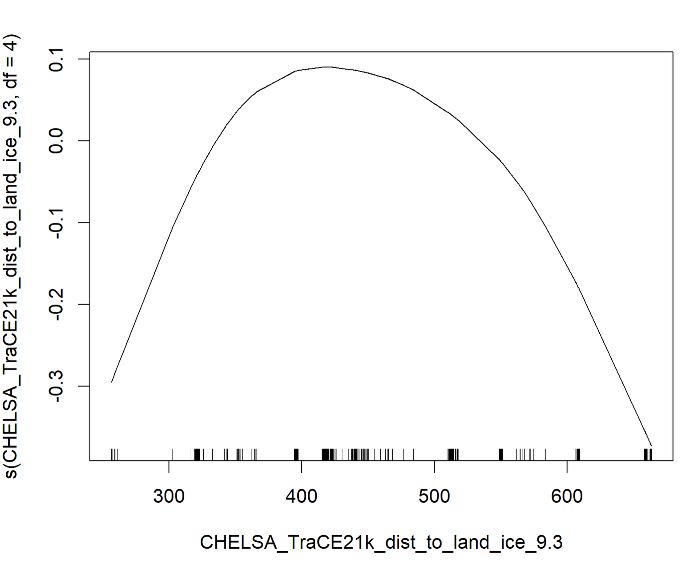 |

**Appendix Figure 1:** Response curves for all predictors used in the GAM model of community-level plant species richness of the Western Siberia Arctic region. Response curves are for **a.** ‘Mean annual paleotemperature’ (12.100 years ago) (°C); **b.** ‘Climate moisture index (max)’ (humidity_penman_max); **c.** ‘Mean january temperature’ (°K); **d**. ‘Mean annual paleoprecipitation’ (17.200 years ago) (mm); **e.** ‘(log transformed) slope’; **f.** ‘Mean daily maximum air temperature of the warmest month (BIO10_05)’ (0.1*°C); **g.** ‘Isothermality’; **h.** ‘Distance to land ice’ (9300 years ago) (km).


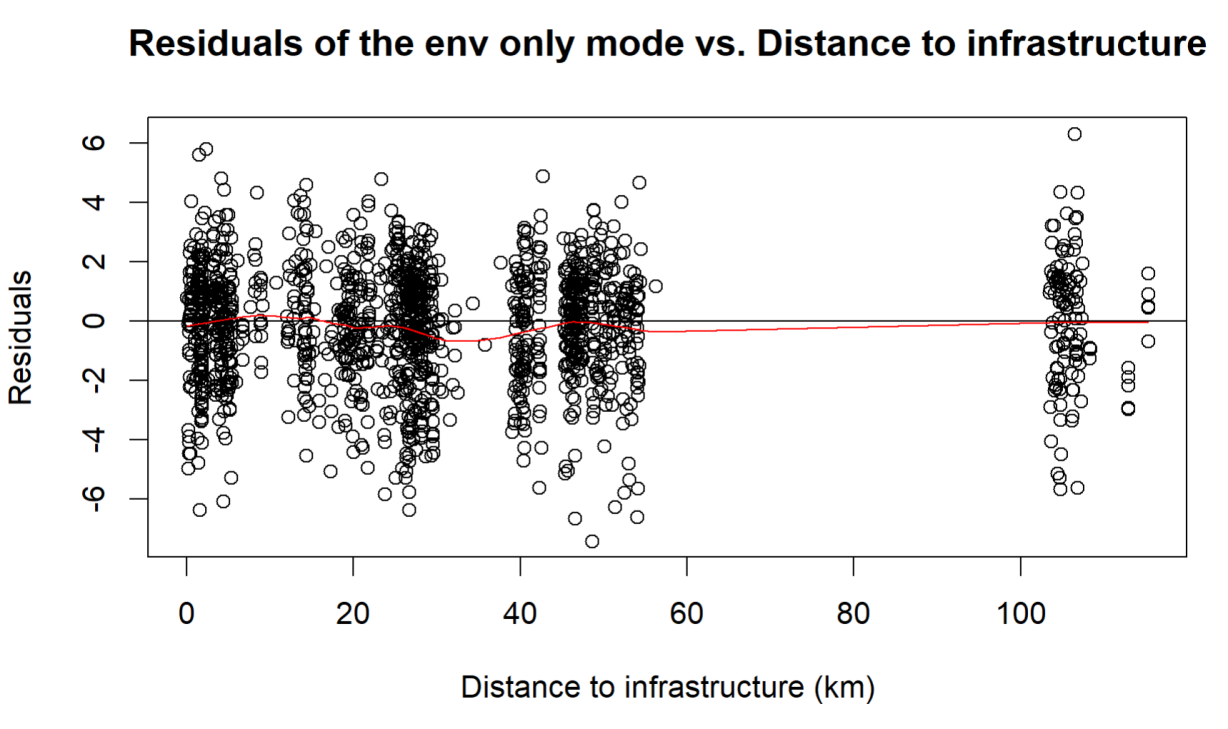


**Appendix Figure 2:** GAM residuals to distance to infrastructure. Loess function (span 0.3) is used to produce a trend line.


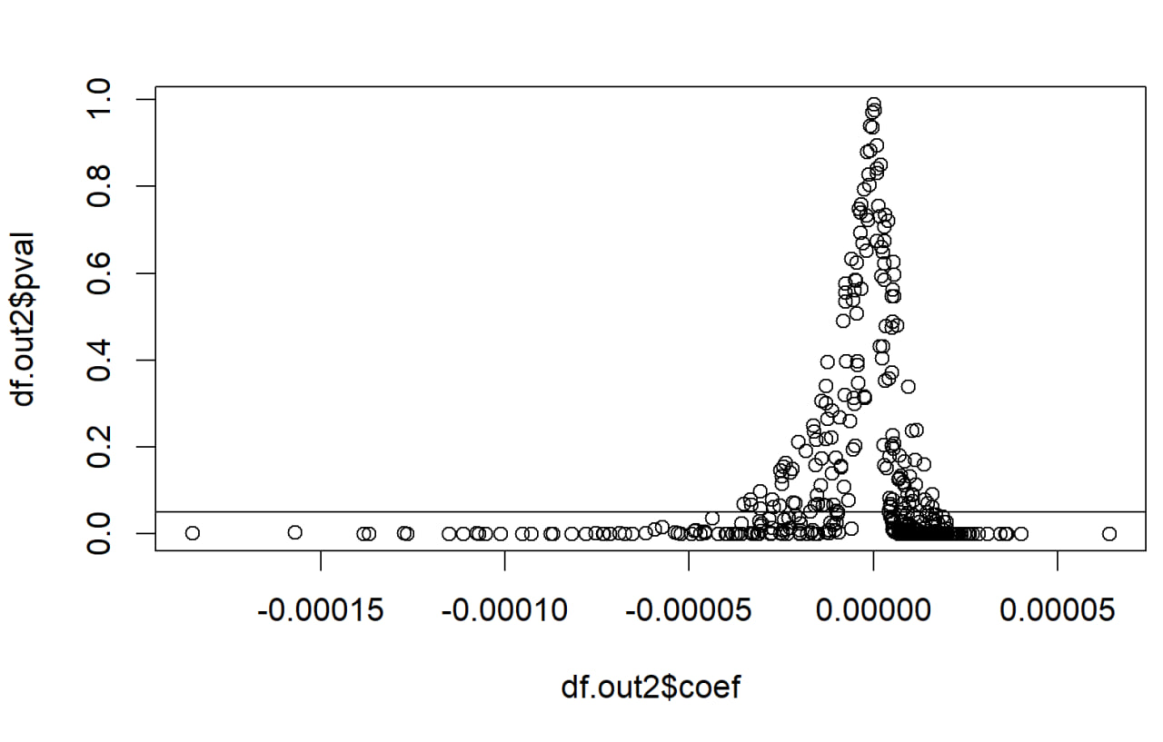


**Appendix Figure 3:** The relationships between the occurrences of each of the 840 species and distance to infrastructure. The x-axis displays the coefficients of logistic regression models and y-axis displays corresponding p-values. The black line is a threshold, separating statistically significant cases from insignificant ones.


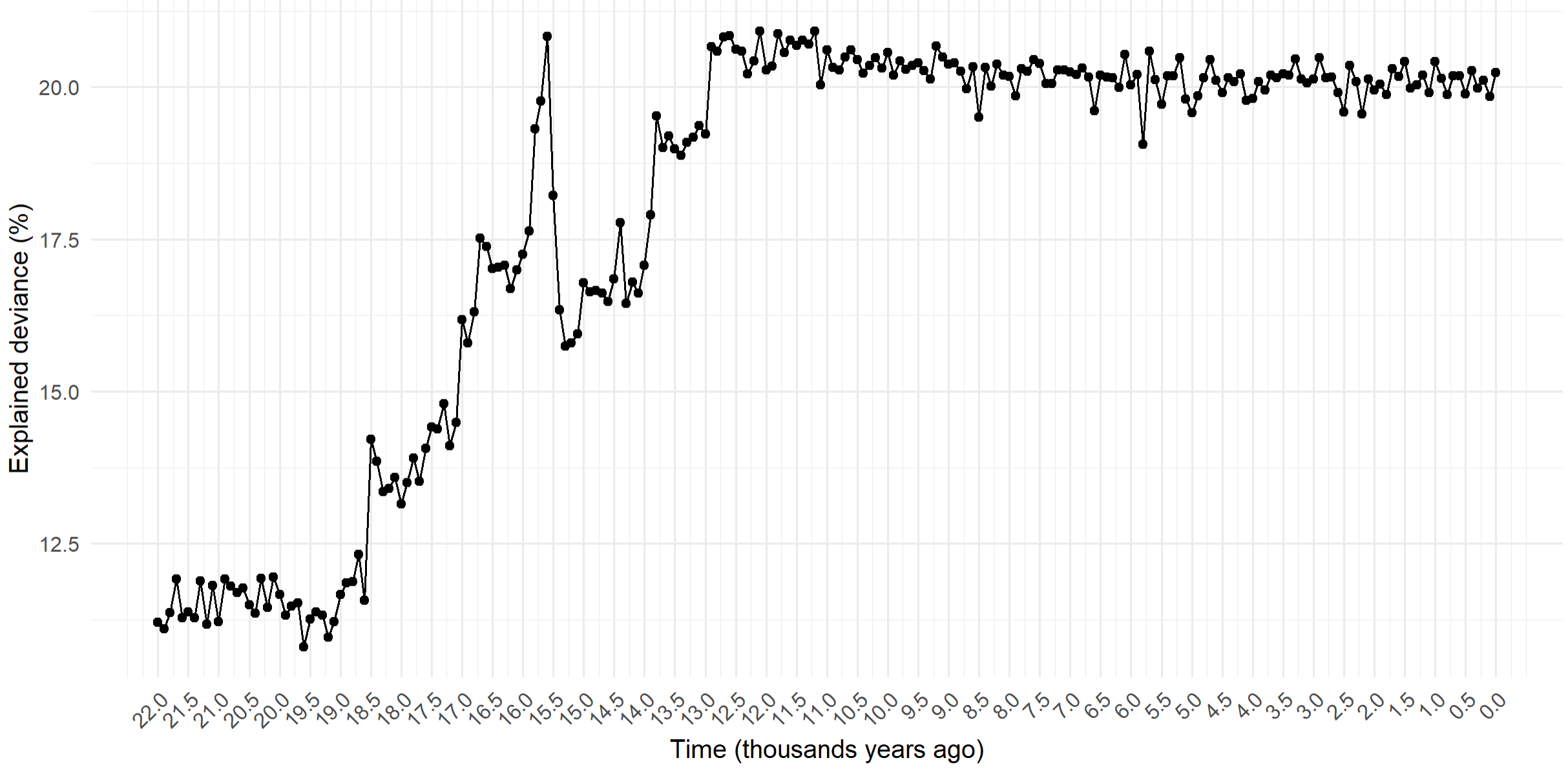


**Appendix Figure 4:** Explained deviance of ‘paleotemperature’ throughout the time since the Last Glacial Maximum (22.000 years ago).

**
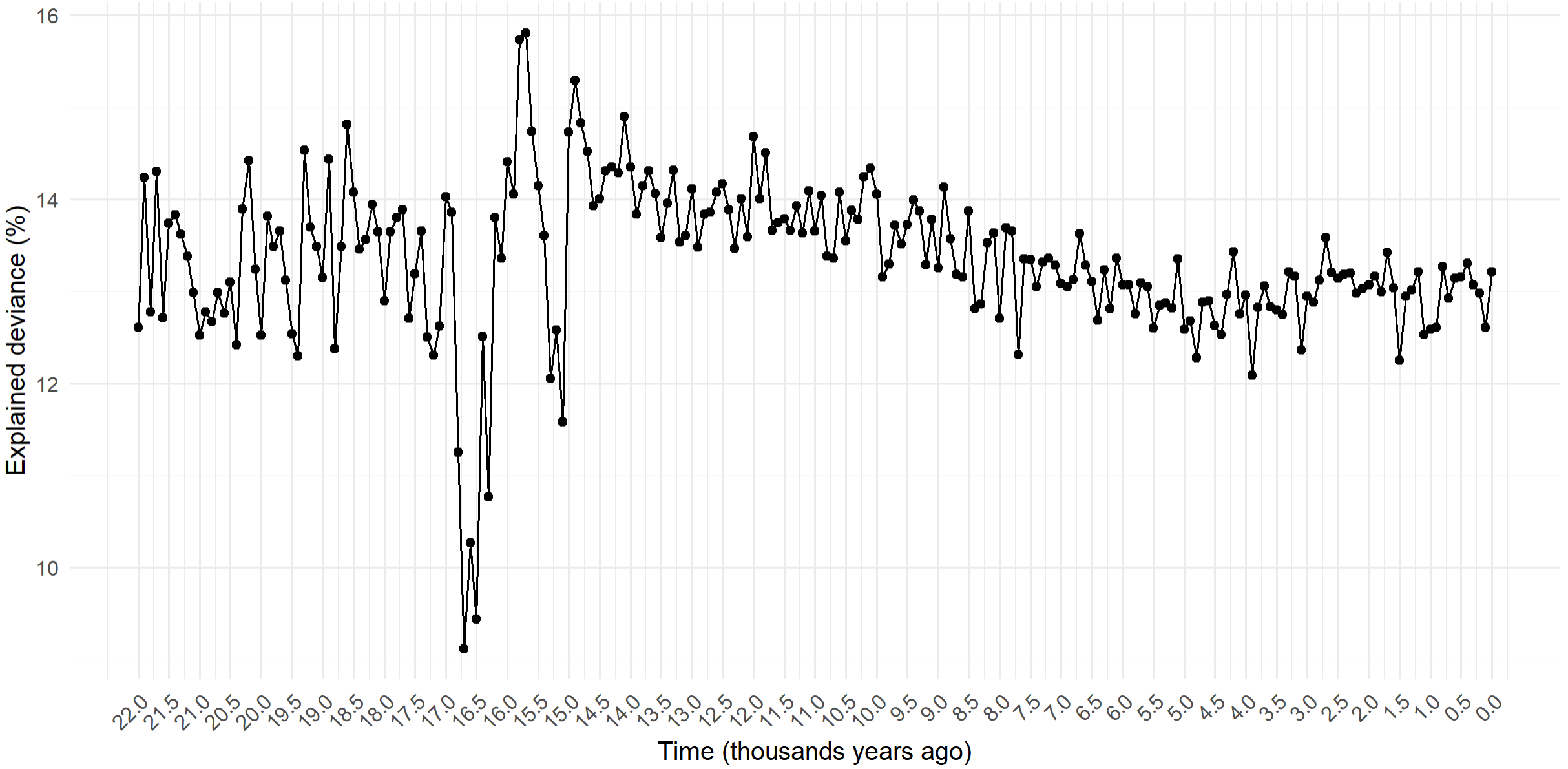
**

**Appendix Figure 5:** Explained deviance of ‘paleoprecipitation’ throughout the time since the Last Glacial Maximum (22.000 years ago).

**
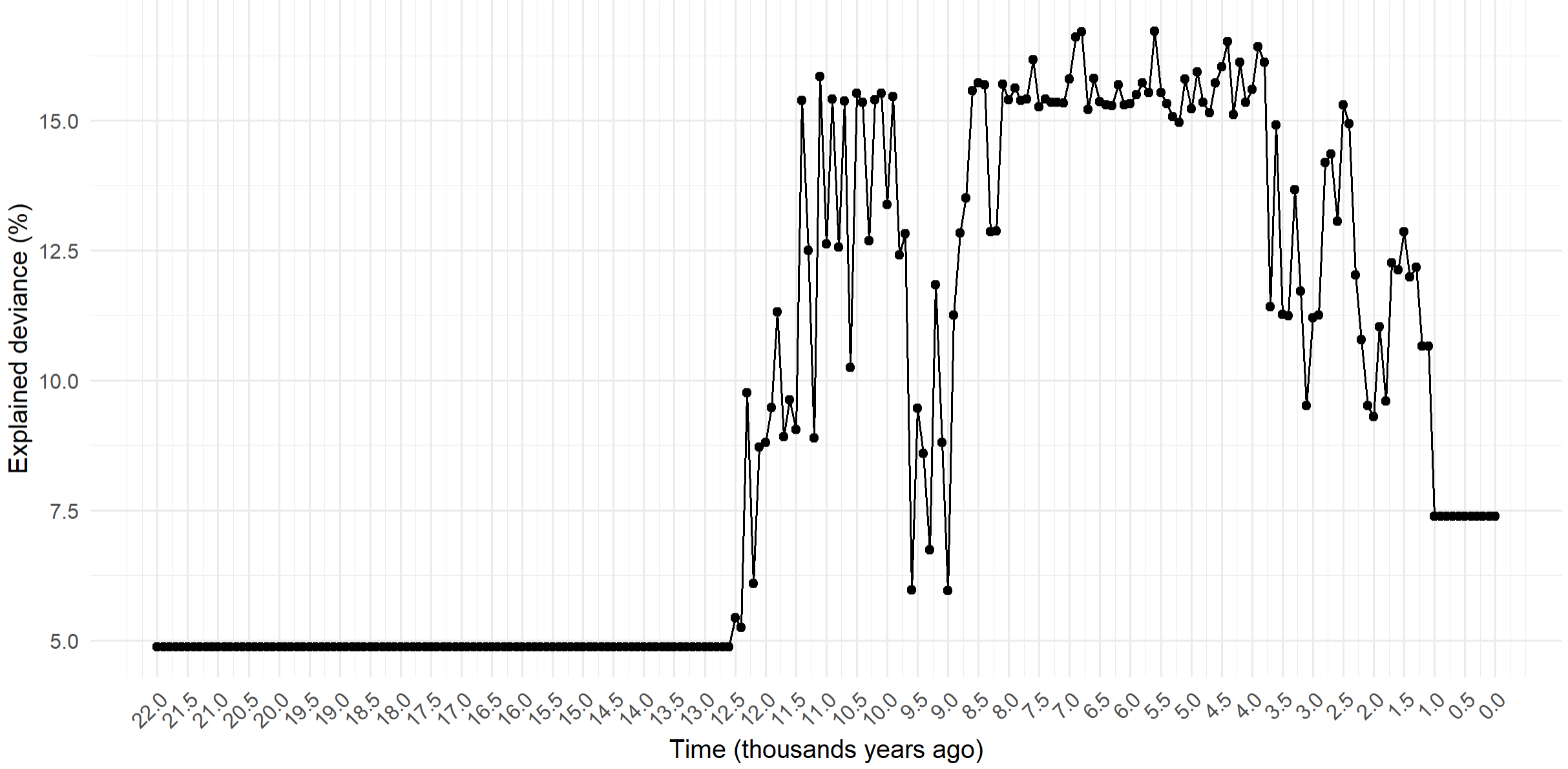
**

**Appendix Figure 6**: Explained deviance of ‘distance to land ice’ predictor throughout the time since the Last Glacial Maximum (22.000 years ago).

**Appendix Table S1:** Full list of tested predictors (green color and bold font shows selected predictors in final model, red color - predictors omitted because of their low predictive power, black color – left out due to high correlation with selected predictors). High correlation (>0.7) with other predictors indicated only for predictors with explained deviance higher than 5%. For paleoclimatic predictors explained deviance is given for the time period 12.100 years ago (highest PP tested) as well as for the time period used in the model.

| **N** | **Predictor** | **Adjusted explained deviance (%)** | **High correlation (>0.7)**  **with other predictors** | **Original resolution** | **Source** |
| --- | --- | --- | --- | --- | --- |
| **1** | **Mean annual temperature (12.100 years ago)** | **21.0** |  | **1000** | **CHELSA TraCE21k dataset (Karger et al., 2021)** |
| 2 | Longitude | 21.0 | Mean January temperature (negative), distance to infrastructure (0.72), temperature seasonality (0.72), climate moisture index range, potential evapotranspiration min (negative) | - | - |
| 3 | Mean monthly precipitation amount of the warmest quarter (bio10_18) | 20.8 | Growing degree days heat sum above 5°C, mean July temperature, MGTM, mean potential evapotranspiration, mean daily maximum air temperature of the warmest month, mean daily mean air temperatures of the warmest quarter (0.74), mean daily mean air temperatures of the wettest quarter, precipitation amount of the wettest month, annual precipitation amount, precipitation seasonality, mean monthly precipitation amount of the warmest quarter; latitude (negative) | 1000 | CHELSA Bioclim (Karger et al., 2016) |
| 4 | Mean annual air temperature (bio10_01) | 20.1 | Mean July temperature, MGTM, mean potential evapotranspiration, mean daily minimum air temperature of the coldest month, mean daily mean air temperatures of the wettest quarter, mean daily mean air temperatures of the coldest quarter, annual precipitation amount, precipitation amount of the wettest month, mean monthly precipitation amount of the wettest quarter, mean monthly precipitation amount of the warmest quarter; latitude (negative) | 1000 | CHELSA Bioclim (Karger et al., 2016) |
| 5 | Mean annual ground temperatures (MGTM) | 18.9 | Mean annual air temperature, mean July temperature, mean potential evapotranspiration, mean daily minimum air temperature of the coldest month, mean daily mean air temperatures of the wettest quarter, mean daily mean air temperatures of the coldest quarter, annual precipitation amount, precipitation amount of the wettest month, mean monthly precipitation amount of the wettest quarter, mean monthly precipitation amount of the warmest quarter; latitude (negative), paleotemperature | 1000 | Global Permafrost project (Obu, et al., 2019) |
| 6 | Mean monthly precipitation amount of the wettest quarter (bio10_16) | 17.9 | Growing degree days heat sum above 5°C, mean July temperature, MGTM, mean potential evapotranspiration, mean daily maximum air temperature of the warmest month, mean daily mean air temperatures of the warmest quarter, mean daily mean air temperatures of the wettest quarter, precipitation amount of the wettest month, annual precipitation amount, precipitation seasonality, mean monthly precipitation amount of the warmest quarter; latitude (negative) | 1000 | CHELSA Bioclim (Karger et al., 2016) |
| 7 | Precipitation amount of the wettest month (bio10_13) | 17.6 | Mean annual air temperature, growing degree days heat sum above 5°C, mean July temperature, MGTM, mean potential evapotranspiration, mean daily maximum air temperature of the warmest month, mean daily minimum air temperature of the coldest month, mean daily mean air temperatures of the warmest quarter, mean daily mean air temperatures of the wettest quarter, annual precipitation amount, precipitation seasonality, mean monthly precipitation amount of the wettest quarter, mean monthly precipitation amount of the warmest quarter | 1000 | CHELSA Bioclim (Karger et al., 2016) |
| 8 | Mean annual precipitation (12.100 years ago) | 17.6 | MGTM, paleo temperature | 1000 | CHELSA TraCE21k dataset (Karger et al., 2021) |
| 9 | Potential evapotranspiration min (pet_penman_min_19 81.2010) | 16.6 | Longitude (negative) | 1000 | CHELSA new (Brun et al., 2022) |
| 10 | Precipitation seasonality (bio10_15) | 16.1 | Precipitation amount of the wettest month, mean monthly precipitation amount of the wettest quarter, mean monthly precipitation amount of the warmest quarter and MGTM (0.7); Mean daily mean air temperatures of the driest quarter (negative) | 1000 | CHELSA Bioclim (Karger et al., 2016) |
| 11 | Latitude | 15.0 | Negative: mean annual air temperature, growing degree days heat sum above 5°C, mean July temperature, MGTM, mean potential evapotranspiration, mean daily maximum air temperature of the warmest month, mean daily mean air temperatures of the wettest quarter, mean daily mean air temperatures of the warmest quarter, annual precipitation amount, precipitation amount of the wettest month, mean monthly precipitation amount of the wettest quarter, mean monthly precipitation amount of the warmest quarter |  | - |
| 12 | Mean July Temperature (K) (Ist_july) | 14.7 | Mean annual air temperature, MGTM, mean potential evapotranspiration, mean daily mean air temperatures of the wettest quarter, annual precipitation amount, precipitation amount of the wettest month, mean monthly precipitation amount of the wettest quarter, mean monthly precipitation amount of the warmest quarter; longitude (negative) |  | MODIS derived  2000-2019 (MOD11A2 MODIS/Terr a Land Surface Temperature /Emissivity 8- Day L3 Global 1km SIN Grid V006 [Data set]) |
| 13 | Mean daily mean air temperatures of the coldest quarter (bio10_11) | 14.1 | Mean annual air temperature, MGTM | 1000 | CHELSA Bioclim (Karger et al., 2016) |
| 14 | **Climate moisture index max (humidity_penman_m ax_1981.2010)** | **14.0** | **Climate moisture index mean** | **1000** | **CHELSA new (Brun et al., 2022)** |
| 15 | Mean daily mean air temperatures of the driest quarter (bio10_09) | 13.9 | Precipitation seasonality (negative) | 1000 | CHELSA Bioclim (Karger et al., 2016) |
| 16 | Mean daily minimum air temperature of the coldest month (bio10_06) | 12.9 | Mean annual air temperature, MGTM, mean daily mean air temperatures of the coldest quarter, precipitation amount of the wettest month | 1000 | CHELSA Bioclim (Karger et al., 2016) |
| 17 | Mean daily mean air temperatures of the wettest quarter (bio10_08) | 12.8 | Mean annual air temperature, growing degree days heat sum above 5°C, mean July temperature, MGTM, mean potential evapotranspiration, mean daily mean air temperatures of the warmest quarter, annual precipitation amount, precipitation amount of the wettest month, mean monthly precipitation amount of the wettest quarter, mean monthly precipitation amount of the warmest quarter; longitude (negative) | 1000 | CHELSA Bioclim (Karger et al., 2016) |
| **18** | **Mean January temperature (K) (Ist_january)** | **12.6** | **Longitude (negative), сlimate moisture index range (0.7)** |  | **MODIS derived 2000-2019 (MOD11A2 MODIS/Terr a Land Surface Temperature /Emissivity 8- Day L3 Global 1km SIN Grid V006 [Data set])** |
| 19 | Annual precipitation amount (bio10_12) | 12.4 | Mean annual air temperature, growing degree days heat sum above 5°C, mean July temperature, MGTM, mean potential evapotranspiration, mean daily maximum air temperature of the warmest month, mean daily mean air temperatures of the wettest quarter, mean daily mean air temperatures of the warmest quarter, precipitation amount of the wettest month, mean monthly precipitation amount of the driest quarter, mean monthly precipitation amount of the wettest quarter, mean monthly precipitation amount of the warmest quarter; latitude (negative) | 1000 | CHELSA Bioclim (Karger et al., 2016) |
| **20** | **Mean annual precipitation (17.200 years ago)** | **12.3** |  | **1000** | **CHELSA TraCE21k dataset (Karger et al.,**  **2021)** |
| 21 | Distance to land ice (12.100 years ago) | 12.1 | MGTM, paleo temperature | 1000 | CHELSA TraCE21k dataset (Karger et al., 2021) |
| 22 | Climate moisture index range (humidity_penman_ra nge_1981.2010) | 11.1 | Longitude, temperature seasonality | 1000 | CHELSA Bioclim (Karger et al., 2016) |
| **23** | **Mean daily maximum air temperature of the warmest month**  **(bio10_05)** | **10.8** | **Growing degree days heat sum above 5°C, mean potential evapotranspiration, mean daily mean air temperatures of the warmest quarter, annual precipitation amount, precipitation amount of the wettest month, mean monthly precipitation amount of the wettest quarter, mean monthly precipitation amount of the warmest quarter, mean monthly precipitation amount of the wettest quarter, mean monthly precipitation amount of the warmest quarter; latitude (negative)** | **1000** | **CHELSA Bioclim (Karger et al., 2016)** |
| **24** | **Distance to infrastructure** | **10.8** | **Longitude** | **-** | **OSM based (https://www. openstreetm ap.org/)** |
| 25 | Growing degree days heat sum above 5°C (gdd_5_1979_2013) | 10.7 | Mean July temperature, mean monthly precipitation amount of the warmest quarter, mean potential evapotranspiration, mean daily maximum air temperature of the warmest month, mean daily mean air temperatures of the warmest quarter, mean daily mean air temperatures of the wettest quarter, mean daily mean air temperatures of the warmest quarter, annual precipitation amount, precipitation amount of the wettest month, mean monthly precipitation amount of the wettest quarter; longitude  (negative) | 1000 | CHELSA new (Brun et al., 2022) |
| **26** | **Isothermality (bio10_03)** | **10.3** | **Cloud area fraction (negative, -0.72)** | **1000** | **CHELSA Bioclim (Karger et al., 2016)** |
| **27** | **Log transformed slope** | **9.6** | **-** | **10** | **ArcticDEM based (Morin et al., 2016)** |
| 28 | Mean potential evapotranspiration (pet_penman_mean_1 981.2010) | 9.3 | MGTM, mean annual air temperature, growing degree days heat sum above 5°C, mean July temperature, mean daily maximum air temperature of the warmest month, mean daily mean air temperatures of the wettest quarter, mean daily mean air temperatures of the warmest quarter, annual precipitation amount, precipitation amount of the wettest month, mean monthly precipitation amount of the wettest quarter, mean monthly precipitation amount of the warmest quarter | 1000 | CHELSA new (Brun et al., 2022) |
| 29 | Mean daily mean air temperatures of the warmest quarter (bio10_10) | 9.3 | Mean monthly precipitation amount of the warmest quarter, latitude, growing degree days heat sum above 5°C , mean, max and range potential evapotranspiration; latitude (negative) | 1000 | CHELSA Bioclim (Karger et al., 2016) |
| 30 | Climate moisture index mean (humidity_penman_m ean_1981.2010) | 9.2 | Climate moisture index max, site water balance | 1000 | CHELSA new (Brun et al., 2022) |
| 31 | Site water balance (swb_1981.2010) | 8.4 | Climate moisture index mean | 1000 | CHELSA new (Brun et al., 2022) |
| 32 | Cloud area fraction (tcc_max_1981.2010) | 7.1 | Isothermality | 1000 | CHELSA new (Brun et al., 2022) |
| **33** | **Distance to land ice (9.300 years ago)** | **6.7** |  | **1000** | **CHELSA TraCE21k dataset (Karger et al.,**  **2021)** |
| 34 | Mean monthly precipitation amount of the driest quarter (bio10_17) | 6.1 | Annual precipitation amount | 1000 | CHELSA Bioclim (Karger et al., 2016) |
| 35 | Standard deviation of altitude | 5.6 | Standard deviation of altitude | 10 | ArcticDEM based (Morin et al., 2016) |
| 36 | Slope | 5.4 | Mean wind speed | 10 | ArcticDEM based (Morin et al., 2016) |
| 37 | Mean wind speed | 5.1 | Slope | 100 | Global Wind Atlas (https://globa lwindatlas.info/) |
| 38 | Temperature seasonality (bio10_04) | 5.1 | Climate moisture index range, longitude, mean daily minimum air temperature of the coldest month, mean daily mean air temperatures of the coldest quarter | 1000 | CHELSA Bioclim (Karger et al., 2016) |
| 39 | Incidence angle raster map | 4.7 |  | 10 | ArcticDEM based (Morin et al., 2016) |
| 40 | Beam irradiance | 3.5 |  | 10 | ArcticDEM based (Morin et al., 2016) |
| 41 | Mean monthly precipitation amount of the coldest quarter  (bio10_19) | 3.3 |  | 1000 | CHELSA Bioclim (Karger et al., 2016) |
| 42 | Mean diurnal air temperature range  (bio10_02) | 3.3 |  | 1000 | CHELSA Bioclim (Karger et al., 2016) |
| 43 | Diffuse irradiance | 3.2 |  | 10 | ArcticDEM based (Morin et al., 2016) |
| 44 | Paleoaltitude | 3 |  | 1000 | CHELSA TraCE21k dataset (Karger et al., 2021) |
| 45 | Global (total) irradiance | 2.7 |  | 10 | ArcticDEM based (Morin et al., 2016) |
| 46 | Precipitation amount of the driest month (bio10_14) | 2.6 |  | 1000 | CHELSA Bioclim (Karger et al., 2016) |
| 46 | Terrain wetness index | 2.6 |  | 1000 | High resolution global topographic index values (Marthews et. al., 2015) |
| 47 | NDVI | 1.8 |  | 1000 | Sentinel derived (JuneAugust 2019-2020) |
| 48 | pet_penman_max_1981.2010 | 1.7 |  | 1000 | CHELSA new (Brun et al., 2022) |
| 49 | Topographic position index | 1.6 |  | 10 | ArcticDEM based (Morin et al., 2016) |
| 50 | pet_penman_range_1 981.2010 | 1.3 |  | 1000 | CHELSA new (Brun et al., 2022) |
| 51 | Annual range of air temperature (bio10_07) | 1.0 |  | 1000 | CHELSA Bioclim (Karger et al., 2016) |
| 52 | Climate moisture index min (humidity_penman_min_1981.2010) | 0.9 |  | 1000 | CHELSA new (Brun et al., 2022) |
| 53 | modcf_intraanualsd_1 | 0.9 |  | 1000 | CHELSA Bioclim (Karger et al., 2016) |
| 54 | Aspect | 0.2 |  | 10 | ArcticDEM based (Morin et al., 2016) |
| 55 | Altitude | 0.0 |  | 10 | ArcticDEM based (Morin et al., 2016) |

**Appendix Table S2:** Full list of species tested for their relationship to distance to infrastructure (green species name color indicates statistically significant positive relationships, blue indicates negative, and yellow cell color signifies species with highly statistically significant relationships to distance from infrastructure), sorted from minimum to maximum p-value. Only species with at least 10 occurrences were included in the test.

| **Species name** | **coefficient** | **p-value** |
| --- | --- | --- |
| ***Sphagnum riparium* Ångstr.** | 0.000064 | <0.000001 |
| ***Andromeda polifolia* s*. pumila* V.M. Vinogr.** | 0.000040 | <0.000001 |
| ***Salix pulchra* Cham.** | 0.000026 | <0.000001 |
| ***Sphenolobus minutus* Schreb. Berggr.** | 0.000025 | <0.000001 |
| ***Ptilidium ciliare* L. Hampe** | 0.000023 | <0.000001 |
| ***Aulacomnium turgidum* Wahlenb. Schwaegr.** | 0.000022 | <0.000001 |
| ***Stellaria longipes* taxon *peduncularis*** | 0.000023 | <0.000001 |
| ***Arctagrostis latifolia* Br. Griseb.** | 0.000021 | <0.000001 |
| ***Cetraria laevigata* Rass.** | 0.000022 | <0.000001 |
| ***Dicranum elongatum* Schleich. ex Schwaegr.** | 0.000017 | <0.000001 |
| ***Dryas punctata* Juz.** | 0.000021 | <0.000001 |
| ***Cladonia chlorophaea* Somm. Spreng.** | 0.000022 | <0.000001 |
| **Unknown liverwort** | 0.000022 | <0.000001 |
| ***Pedicularis labradorica* Wirsing** | 0.000034 | <0.000001 |
| ***Valeriana capitata* Pall. ex Link** | 0.000019 | <0.000001 |
| ***Sphagnum balticum* Russ. C. Jens.** | 0.000021 | <0.000001 |
| ***Calamagrostis holmii* Lange** | 0.000017 | <0.000001 |
| ***Minuartia macrocarpa* Pursh. Ostenf.** | 0.000026 | <0.000001 |
| ***Vaccinium vitis.idaea* s. *minus* Lodd. Hultén** | 0.000015 | <0.000001 |
| ***Rhododendron tomentosum* s. *decumbens* Aiton. Elven. D.F. Murray** | 0.000015 | <0.000001 |
| ***Micranthes nelsoniana* D. Don. Small** | 0.000018 | <0.000001 |
| ***Tomentypnum nitens* Hedw. Loeske** | 0.000018 | <0.000001 |
| ***Carex concolor* R. Br.** | 0.000015 | <0.000001 |
| ***Dactylina arctica* Richardson. Nyl.** | 0.000014 | <0.000001 |
| ***Eriophorum angustifolium* Honck.** | 0.000014 | <0.000001 |
| ***Eriophorum vaginatum* L.** | 0.000015 | <0.000001 |
| ***Pedicularis oederi* Vahl** | 0.000017 | <0.000001 |
| ***Tofieldia coccinea* Richardson** | 0.000029 | <0.000001 |
| ***Myosotis alpestris* s. *asiatica* Vestergr.** | 0.000019 | <0.000001 |
| ***Festuca brachyphylla* Schult.** | 0.000036 | <0.000001 |
| ***Pedicularis interior* Hultén. Molau. D.F. Murray** | 0.000021 | <0.000001 |
| ***Lagotis glauca* s. *minor* Willd Hultén** | 0.000015 | <0.000001 |
| ***Luzula kjellmaniana* Miyabe. Kudô** | 0.000020 | <0.000001 |
| ***Sphagnum squarrosum* Crome** | 0.000019 | <0.000001 |
| ***Dactylina ramulosa* Hook. Tuck.** | 0.000036 | <0.000001 |
| ***Poa arctica* R. Br.** | 0.000013 | <0.000001 |
| ***Eriophorum tolmatchevii* M.S. Novos.** | 0.000021 | <0.000001 |
| ***Asahinea chrysantha* Tuck. Culb. C.Culb.** | 0.000023 | <0.000001 |
| ***Poa arctica* s. *arctica* R. Br.** | 0.000025 | <0.000001 |
| ***Ranunculus subborealis* s. *subborealis* Tzvelev** | 0.000015 | <0.000001 |
| ***Salix lanata* v. *lanata* L.** | 0.000012 | <0.000001 |
| ***Polytrichum juniperinum* Hedw.** | 0.000012 | <0.000001 |
| ***Pedicularis lapponica* L.** | 0.000016 | <0.000001 |
| ***Cladonia pleurota* Flörke. Schaer.** | 0.000014 | <0.000001 |
| ***Saxifraga hirculus* L.** | 0.000021 | 0.000001 |
| ***Cladonia gracilis* s. lat. L. Willd.** | 0.000010 | 0.000001 |
| ***Sphagnum capillifolium* Ehrh. Hedw.** | 0.000036 | 0.000001 |
| ***Parrya nudicaulis* L. Regel** | 0.000016 | 0.000001 |
| ***Dicranum spadiceum* J. E. Zetterst.** | 0.000010 | 0.000003 |
| ***Lloydia serotina* L. Rchb.** | 0.000018 | 0.000003 |
| ***Distichium capillaceum* Hedw. Bruch. Schimp.** | 0.000024 | 0.000003 |
| ***Ditrichum flexicaule* Schwaegr. Hampe** | 0.000020 | 0.000004 |
| ***Sanionia uncinata* Hedw. Loeske** | 0.000010 | 0.000004 |
| ***Eutrema edwardsii* R. Br.** | 0.000019 | 0.000006 |
| ***Cladonia cornuta* L. Hoffm.** | 0.000017 | 0.000007 |
| ***Bryum species*** | 0.000031 | 0.000007 |
| ***Polytrichum hyperboreum* R. Br.** | 0.000010 | 0.000010 |
| ***Pertusaria dactylina* Ach. Nyl.** | 0.000018 | 0.000011 |
| ***Cardamine polemonioides* Rouy** | 0.000017 | 0.000012 |
| ***Salix myrtilloides* L.** | 0.000016 | 0.000012 |
| ***Vaccinium uliginosum* s. *microphyllum* Lange. Tolm.** | 0.000009 | 0.000013 |
| ***Pedicularis verticillata* L.** | 0.000019 | 0.000014 |
| ***Pleurozium schreberi* Willdenow ex Brid. Mitt.** | 0.000011 | 0.000014 |
| ***Salix reptans* Rupr.** | 0.000012 | 0.000017 |
| ***Pohlia cruda* Hedw. Lindb.** | 0.000020 | 0.000018 |
| ***Warnstorfia exannulata* Schimp. Loeske** | 0.000017 | 0.000022 |
| ***Peltigera didactyla* s. lat. With. Laundon** | 0.000027 | 0.000025 |
| ***Huperzia arctica* Grossh. ex Tolm. Sipliv.** | 0.000017 | 0.000026 |
| ***Eriophorum brachyantherum* Trautv. C.A. Mey.** | 0.000023 | 0.000033 |
| ***Polytrichum jensenii* I. Hagen** | 0.000014 | 0.000034 |
| ***Poa pratensis* s. *alpigena* Lindm. Hiitonen** | 0.000011 | 0.000042 |
| ***Hedysarum hedysaroides* s. *arcticum* B. Fedtsch. P.W. Ball** | 0.000019 | 0.000042 |
| ***Cladonia stygia* Fr. Ruoss** | 0.000009 | 0.000055 |
| ***Limprichtia revolvens* Swartz. Loeske** | 0.000017 | 0.000060 |
| ***Pyrola grandiflora* s. *norvegica* Knaben. A. Löve D. Löve** | 0.000019 | 0.000077 |
| ***Sphagnum russowii* Warnst.** | 0.000023 | 0.000185 |
| ***Cerastium regelii* taxon *jenisejense* Hultén.** | 0.000026 | 0.000196 |
| ***Aulacomnium* *palustre* Hedw. Schwaegr.** | 0.000008 | 0.000198 |
| ***Cerastium* *regelii* Ostenf.** | 0.000017 | 0.000200 |
| ***Alopecurus* *borealis* Trin.** | 0.000009 | 0.000216 |
| ***Calliergon cordifolium* Hedw. Kindb.** | 0.000019 | 0.000223 |
| ***Drepanocladus species*** | 0.000018 | 0.000246 |
| ***Alectoria ochroleuca* Hoffm. Massal.** | 0.000008 | 0.000289 |
| ***Carex bigelowii* s. *ensifolia* Turcz. ex Ledeb. ined.** | 0.000007 | 0.000345 |
| ***Plagiomnium* *ellipticum* Brid. T. Kop.** | 0.000016 | 0.000350 |
| ***Petasites frigidus* L. Fr.** | 0.000009 | 0.000434 |
| ***Micranthes hieraciifolia* Waldst. Kit. ex Willd. Haw.** | 0.000015 | 0.000488 |
| ***Hylocomium splendens* Hedw. Schimp.** | 0.000007 | 0.000595 |
| ***Cetraria islandica* L. Ach.** | 0.000007 | 0.000611 |
| ***Sphagnum fimbriatum* Wils.** | 0.000017 | 0.000623 |
| ***Arctocetraria andrejevii* Oxner. Kärnefelt Thell** | 0.000012 | 0.000771 |
| ***Chrysosplenium alternifolium* L.** | 0.000018 | 0.001110 |
| ***Carex rariflora* Wahlenb Sm.** | 0.000009 | 0.001133 |
| ***Dryas species*** | 0.000018 | 0.001266 |
| ***Bistorta vivipara* L. Delarbre** | 0.000007 | 0.001282 |
| ***Cladonia cyanipes* Sommerf. Nyl.** | 0.000019 | 0.001299 |
| ***Rhytidium rugosum* Ehrh. ex Hedw. Kindb.** | 0.000012 | 0.001406 |
| ***Cerastium maximum* L.** | 0.000018 | 0.001460 |
| ***Micranthes nivalis* L. Small** | 0.000016 | 0.001529 |
| ***Astragalus alpinus* L.** | 0.000018 | 0.001622 |
| ***Peltigera membranacea* Ach. Nyl.** | 0.000012 | 0.002161 |
| ***Sphagnum compactum* Lam. DC.** | 0.000014 | 0.002175 |
| ***Coptidium lapponicum* L. Tzvelev** | 0.000014 | 0.002511 |
| ***Festuca ovina* s. *ovina*. L.** | 0.000008 | 0.002666 |
| ***Plagiomnium species*** | 0.000019 | 0.002745 |
| ***Caltha palustris* s. *radicans* T.F. Forst. Syme** | 0.000013 | 0.002790 |
| ***Chrysosplenium alternifolium* s. *sibiricum* Ser. ex DC. Hultén** | 0.000019 | 0.002910 |
| ***Pedicularis albolabiata* Hultén. Kozhevn.** | 0.000013 | 0.003102 |
| ***Dupontia fisheri* R. Br.** | 0.000013 | 0.003189 |
| ***Trisetum sibiricum* s. *litorale* Rupr. ex Roshev.** | 0.000018 | 0.003714 |
| ***Cladonia subfurcata* NyL. Arn.** | 0.000009 | 0.003821 |
| ***Rumex arcticus* Trautv.** | 0.000011 | 0.004952 |
| ***Racomitrium lanuginosum* Hedw. Brid.** | 0.000007 | 0.005480 |
| ***Nephroma expallidum* NyL. Nyl.** | 0.000008 | 0.005518 |
| ***Juncus biglumis* L.** | 0.000016 | 0.005531 |
| ***Cladonia arbuscula* s. lat. Wallr. Hale. W.Culb.** | 0.000006 | 0.005653 |
| ***Luzula confusa* Lindeb.** | 0.000006 | 0.005955 |
| ***Flavocetraria cucullata* Bell. Kärnefelt. Thell** | 0.000005 | 0.006498 |
| ***Ranunculus nivalis* L.** | 0.000015 | 0.007538 |
| ***Ranunculus monophyllus* Ovcz. s. lat.** | 0.000017 | 0.007907 |
| ***Orthocaulis binsteadii* Kaal. H.Buch** | 0.000012 | 0.008714 |
| ***Campylium polygamum* Schimp. C. Jens.** | 0.000019 | 0.009001 |
| ***Poa alpina* L.** | 0.000012 | 0.009554 |
| ***Pyrola grandiflora* Radius** | 0.000009 | 0.009929 |
| ***Bistorta elliptica* Willd. ex Spreng. Kom. ex V.V. Petrovsky** | 0.000008 | 0.010558 |
| ***Saxifraga cernua* L.** | 0.000009 | 0.010613 |
| ***Carex chordorrhiza* Ehrh. ex L. f.** | 0.000012 | 0.010776 |
| ***Betula nana* L.** | 0.000005 | 0.011160 |
| ***Gymnomitrion corallioides* Nees** | 0.000011 | 0.011695 |
| ***Brachythecium albicans* Hedw. B.S.G.** | 0.000020 | 0.013121 |
| ***Astragalus alpinus* v. *arcticus* Sond.n. Lindm.** | 0.000011 | 0.013921 |
| ***Salix polaris* Wahlenb.** | 0.000005 | 0.014178 |
| ***Carex rotundata* Wahlenb.** | 0.000007 | 0.014638 |
| ***Sphagnum rubellum* Wils.** | 0.000014 | 0.014707 |
| ***Lobaria linita* Ach. Rabenh.** | 0.000009 | 0.016445 |
| ***Lophozia ventricosa* v. *longiflora* Nees. Macoun** | 0.000016 | 0.018996 |
| ***Hypnum species*** | 0.000010 | 0.021583 |
| ***Vaccinium uliginosum* L.** | 0.000008 | 0.021780 |
| ***Drepanocladus aduncus* Hedw. Warnst.** | 0.000018 | 0.024675 |
| ***Tritomaria quinquedentata* Huds. H.Buch** | 0.000010 | 0.025226 |
| ***Rubus chamaemorus* L.** | 0.000005 | 0.026845 |
| ***Nephroma arcticum* L. Torss.** | 0.000012 | 0.027251 |
| ***Cladonia subcervicornis* Vain. Kernst.** | 0.000020 | 0.027609 |
| ***Pohlia nutans* Hedw. Lindb.** | 0.000006 | 0.027707 |
| ***Mnium blyttii* Bruch. Schimp.** | 0.000019 | 0.027902 |
| ***Claytonia species*** | 0.000017 | 0.029141 |
| ***Polytrichum piliferum* Hedw.** | 0.000007 | 0.029689 |
| ***Equisetum arvense* L.** | 0.000005 | 0.031055 |
| ***Lophoziopsis polaris* R. M. Schust. Konstant. Vilnet** | 0.000016 | 0.034495 |
| ***Gowardia nigricans* Ach. P.Halonen** | 0.000005 | 0.037191 |
| ***Cladonia deformis* L. Hoffm.** | 0.000012 | 0.037892 |
| ***Cetrariella delisei* Bory ex Schaer. Kärnefelt et A. Thell** | 0.000005 | 0.040208 |
| ***Flavocetraria nivalis* L. Kärnefelt. Thell** | 0.000004 | 0.040543 |
| ***Veratrum album* s. *misae* Sirj. Tzvelev** | 0.000019 | 0.040585 |
| ***Salix reticulata* L.** | 0.000014 | 0.042406 |
| ***Pertusaria panyrga* Ach. A. Massal.** | 0.000011 | 0.044404 |
| ***Coptidium pallasii* Schltdl. Tzvelev** | 0.000016 | 0.045090 |
| ***Timmia austriaca* Hedw.** | 0.000017 | 0.045125 |
| ***Calamagrostis neglecta* s. *neglecta* Ehrh. P. Gaertn.** | 0.000009 | 0.048253 |
| ***Festuca rubra* s. *richardsonii* Hook. Hultén** | -0.000037 | <0.000001 |
| ***Calamagrostis neglecta* Ehrh. P. Gaertn.** | -0.000072 | <0.000001 |
| ***Andromeda polifolia* L.** | -0.000052 | <0.000001 |
| ***Calamagrostis species*** | -0.000038 | <0.000001 |
| ***Ranunculus subborealis* Tzvelev** | -0.000105 | <0.000001 |
| ***Polytrichastrum alpinum* v. *fragile* Bryhn. D. G. Long. Hedw. G. L. Sm.** | -0.000088 | <0.000001 |
| ***Cladonia species*** | -0.000028 | <0.000001 |
| ***Polytrichum strictum* Menzies ex Brid.** | -0.000015 | <0.000001 |
| ***Carex bigelowii* Torr.** | -0.000137 | <0.000001 |
| ***Stellaria crassifolia* Ehrh.** | -0.000068 | <0.000001 |
| ***Cladonia mitis* Sandst.** | -0.000111 | <0.000001 |
| ***Vaccinium vitis-idaea* L.** | -0.000020 | <0.000001 |
| ***Rhododendron tomentosum* s. *tomentosum* Stokes. Harmaja** | -0.000093 | 0.000001 |
| ***Leptobryum pyriforme* Hedw. Wils.** | -0.000107 | 0.000001 |
| ***Poa alpina* v. *vivipara* L.** | -0.000138 | 0.000002 |
| ***Veratrum album* L.** | -0.000036 | 0.000003 |
| ***Puccinellia sibirica* Holmb.** | -0.000127 | 0.000004 |
| ***Poa species*** | -0.000052 | 0.000006 |
| ***Arctous alpina* L. Nied.** | -0.000016 | 0.000007 |
| ***Carex aquatilis* Wahlenb.** | -0.000038 | 0.000011 |
| ***Lophozia ventricosa* Dicks. Dumort.** | -0.000073 | 0.000023 |
| ***Dicranum brevifolium* Lindb. Lindb.** | -0.000066 | 0.000037 |
| ***Pohlia proligera* Kindb. Broth.** | -0.000087 | 0.000074 |
| ***Oxycoccus microcarpus* Turcz. ex Rupr.** | -0.000115 | 0.000192 |
| ***Salix hastata* L.** | -0.000042 | 0.000239 |
| ***Tetraplodon mnioides* Swartz ex Hedw. Bruch. Schimp.** | -0.000040 | 0.000247 |
| ***Cladonia fimbriata* L. Fr.** | -0.000078 | 0.000253 |
| ***Salix phylicifolia* L.** | -0.000018 | 0.000265 |
| ***Dicranella crispa* Hedw. Schimp.** | -0.000082 | 0.000315 |
| **Unknown lichen crustose** | -0.000073 | 0.000338 |
| ***Oxytropis sordida* Willd. Pers.** | -0.000047 | 0.000380 |
| ***Pedicularis species*** | -0.000032 | 0.000390 |
| ***Dicranum acutifolium* Lindb.. H. Arn. C. Jens.** | -0.000025 | 0.000396 |
| ***Cerastium species*** | -0.000040 | 0.000405 |
| ***Armeria maritima* s. *maritima*. Mill. Willd.** | -0.000025 | 0.000571 |
| ***Oncophorus virens* Hedw. Brid.** | -0.000031 | 0.000647 |
| ***Rubus arcticus* L.** | -0.000033 | 0.000786 |
| ***Cetrariella fastigiata* Bory ex Schaerer. Kärnefelt et A. Thell** | -0.000023 | 0.000800 |
| ***Cladonia decorticata* Flörke. Sprengel** | -0.000101 | 0.001131 |
| ***Deschampsia brevifolia* R. Br.** | -0.000095 | 0.001202 |
| ***Tripleurospermum maritimum* s. *phaeocephalum* Rupr. Hämet. Ahti** | -0.000050 | 0.001233 |
| ***Aulacomnium species*** | -0.000028 | 0.001499 |
| ***Peltigera scabrosa* Th. Fr.** | -0.000012 | 0.001537 |
| ***Solidago virgaurea* s. *lapponica* With. Tzvelev** | -0.000046 | 0.001566 |
| ***Festuca species*** | -0.000033 | 0.001636 |
| ***Polytrichum commune* Hedw.** | -0.000025 | 0.001646 |
| ***Cladonia borealis* Stenroos** | -0.000053 | 0.001875 |
| ***Ochrolechia inaequatula* Nyl. Zahlbr.** | -0.000185 | 0.001952 |
| ***Syntrichia ruralis* Hedw. Web.. D. Mohr** | -0.000069 | 0.001988 |
| ***Pedicularis arctoeuropaea* Hultén. Molau. D.F. Murray** | -0.000076 | 0.002088 |
| ***Cladonia macrophylla* Schaerer. Stenh.** | -0.000062 | 0.002208 |
| ***Geranium krylovii* Tzvelev** | -0.000108 | 0.002718 |
| ***Ceratodon purpureus* Hedw. Brid.** | -0.000020 | 0.003016 |
| ***Amblystegium species*** | -0.000032 | 0.003072 |
| ***Hypogymnia physodes* L. Nyl.** | -0.000127 | 0.003080 |
| ***Tephroseris atropurpurea* Ledeb. Holub** | -0.000013 | 0.003179 |
| ***Chamaedaphne calyculata* L. Moench** | -0.000157 | 0.003283 |
| ***Polemonium boreale* Adams** | -0.000017 | 0.004011 |
| ***Arctocetraria nigricascens* Nyl. Elenkin** | -0.000054 | 0.004223 |
| ***Tanacetum bipinnatum* L. Sch.Bip.** | -0.000009 | 0.004532 |
| ***Equisetum pratense* Ehrh.** | -0.000046 | 0.005474 |
| ***Dicranum scoparium* Hedw.** | -0.000049 | 0.006865 |
| ***Antennaria dioica* L. Gaertn.** | -0.000031 | 0.008018 |
| ***Juncus trifidus* L.** | -0.000031 | 0.008097 |
| ***Eremogone polaris* Schischk. Ikonn.** | -0.000020 | 0.008372 |
| ***Equisetum arvense* s. *alpestre* Wahlenb. Schönswetter. Elven** | -0.000011 | 0.008609 |
| ***Alnus viridis* s. *fruticosa* Rupr. Nyman** | -0.000017 | 0.008644 |
| ***Pohlia atropurpurea* Wahlenb. H. Lindb.** | -0.000048 | 0.009293 |
| ***Parnassia palustris* s. *neogaea* Fernald. Hultén** | -0.000025 | 0.009871 |
| ***Campanula rotundifolia* L.** | -0.000012 | 0.009974 |
| ***Trichophorum cespitosum* s. *cespitosum*. L. Schur** | -0.000059 | 0.010083 |
| ***Gymnomitrion species*** | -0.000024 | 0.011222 |
| ***Salix nummularia* Andersson** | -0.000006 | 0.013053 |
| ***Stereocaulon glareosum* Sav. Magn.** | -0.000028 | 0.013632 |
| ***Kiaeria glacialis* Berggren. I. Hagen** | -0.000023 | 0.014158 |
| ***Pertusaria geminipara* Th. Fr. C.Knight ex Brodo** | -0.000057 | 0.015902 |
| **Unknown liverworts leafy** | -0.000031 | 0.020045 |
| ***Polystichum species*** | -0.000011 | 0.021009 |
| ***Racomitrium species*** | -0.000020 | 0.023916 |
| ***Dicranella subulata* Hedw. Schimp.** | -0.000030 | 0.023974 |
| ***Luzula species*** | -0.000036 | 0.024911 |
| ***Pachypleurum alpinum* Ledeb.** | -0.000010 | 0.025617 |
| ***Sphagnum warnstorfii* Russ.** | -0.000012 | 0.026731 |
| ***Hamatocaulis lapponicus* Norrlin. Hedenas** | -0.000031 | 0.029834 |
| ***Schljakovia kunzeana* Huebener. Konstant.. Vilnet** | -0.000024 | 0.033616 |
| ***Diapensia lapponica* L.** | -0.000028 | 0.035730 |
| ***Calamagrostis neglecta* s. *groenlandica* Schrank. Matuszk.** | -0.000020 | 0.035887 |
| ***Bromopsis pumpelliana* Scribn. Holub** | -0.000023 | 0.036570 |
| ***Cladonia bacilliformis* Nyl. Glück** | -0.000044 | 0.037426 |
| ***Brachythecium salebrosum* Hoffm. ex Web.. D. Mohr. B.S.G.** | -0.000021 | 0.044059 |
| ***Micranthes foliolosa* R. Br. Gornall** | -0.000010 | 0.045560 |
| ***Bryum pseudotriquetrum* Hedw. P.G. Gaertn.** | 0.000007 | 0.050917 |
| ***Pohlia species*** | 0.000014 | 0.051182 |
| ***Antennaria villifera* Boriss.** | -0.000010 | 0.051236 |
| ***Psoroma hypnorum* Vahl. S.F.Gray** | -0.000017 | 0.051353 |
| ***Bryocaulon divergens* Ach. Kärnefelt** | 0.000004 | 0.051853 |
| ***Hamatocaulis vernicosus* Mitt. Hedenas** | -0.000010 | 0.053949 |
| ***Peltigera rufescens* Weis. Humb.** | 0.000008 | 0.058900 |
| *Hieracium alpinum* aggregate | -0.000031 | 0.059313 |
| *Lophozia wenzelii* Nees. Steph. | 0.000016 | 0.062405 |
| *Cladonia ecmocyna* Leight. | -0.000027 | 0.062767 |
| *Hierochloe alpina* Sw. Roem.. Schult. | 0.000005 | 0.063017 |
| *Ochrolechia frigida* Sw. Lynge | 0.000005 | 0.064523 |
| *Paludella squarrosa* Hedw. Brid. | -0.000016 | 0.065622 |
| *Dianthus repens* Willd. | -0.000013 | 0.065724 |
| *Elymus species* | -0.000025 | 0.066234 |
| *Sphagnum lindbergii* Schimp. | -0.000033 | 0.066863 |
| *Cerastium arvense* L. | -0.000014 | 0.067567 |
| *Brachythecium mildeanum* Schimp. Schimp. | -0.000011 | 0.067591 |
| *Crepis tectorum* s. *nigrescens* Pohle. P.D. Sell | -0.000035 | 0.069089 |
| *Sphagnum species* | -0.000015 | 0.069463 |
| *Peltigera aphthosa* L. Willd. | 0.000004 | 0.070123 |
| *Draba glabella* Pursh | 0.000015 | 0.070606 |
| *Oncophorus compactus* B.S.G. Kindb. | -0.000021 | 0.070961 |
| *Cladonia furcata* Huds. Schrad. | 0.000010 | 0.071879 |
| *Peltigera canina* L. Willd. | 0.000008 | 0.071916 |
| *Lycopodium annotinum* s. *alpestre* Hartm. A. Löve. D. Löve | -0.000022 | 0.072934 |
| *Sphagnum Ångstroemii* C. Hart. | 0.000011 | 0.075945 |
| *Calliergon stramineum* Dicks. ex Brid. Kindb. | -0.000007 | 0.077468 |
| *Stereocaulon alpinum* Laur. | 0.000005 | 0.079033 |
| *Milium species* | -0.000033 | 0.079783 |
| *Limprichtia cossonii* Schimp. L.E. Anders. | 0.000014 | 0.079949 |
| *Gymnocolea inflata* Huds. Dumort. | -0.000028 | 0.080068 |
| *Cladonia coccifera* s. lat. L. Willd. | 0.000004 | 0.083402 |
| *Loeskypnum badium* C.J. Hart. Paul | 0.000011 | 0.090222 |
| *Ochrolechia species* | -0.000015 | 0.090407 |
| *Brachythecium species* | 0.000016 | 0.091071 |
| *Polytrichastrum species* | 0.000010 | 0.091463 |
| *Hierochloe pauciflora* R. Br. | 0.000009 | 0.092453 |
| *Rinodina turfacea* Wahlenb. Körb. | -0.000031 | 0.097811 |
| *Aconogonon ochreatum* L. H. Hara | -0.000008 | 0.108972 |
| *Pogonatum urnigerum* Hedw. P. Beauv. | -0.000014 | 0.112405 |
| *Deschampsia sukatschewii* s. *borealis* Trautv. Tzvelev | 0.000008 | 0.112773 |
| *Cinclidium subrotundum* Lindb. | 0.000012 | 0.114097 |
| *Japewia tornoënsis* Nyl. Tønsberg | -0.000025 | 0.115889 |
| *Luzula nivalis* Laest. Spreng. | 0.000008 | 0.118885 |
| *Sphagnum lenense* H. Lindb. ex L.I. Savicz | 0.000007 | 0.126682 |
| *Carex lachenalii* Schkuhr | 0.000007 | 0.130077 |
| *Warnstorfia sarmentosa* Wahlenb. Hedenas | 0.000010 | 0.133161 |
| *Cladonia grayi* G. Merr. ex Sandst. | -0.000025 | 0.133550 |
| *Blepharostoma trichophyllum* Linn. Dumortier | 0.000007 | 0.134934 |
| *Trisetum species* | -0.000011 | 0.139786 |
| *Hypogymnia subobscura* Vainio. Poelt | -0.000023 | 0.141518 |
| *Salix rosmarinifolia* L. | -0.000025 | 0.146787 |
| *Larix species* | -0.000022 | 0.149898 |
| *Cladonia uncialis* L. Wigg. | 0.000003 | 0.151533 |
| *Artemisia borealis* Pall. | -0.000009 | 0.153406 |
| *Icmadophila ericetorum* L. Zahlbr. | -0.000025 | 0.156271 |
| *Cladonia pyxidata* L. Hoffm. | -0.000009 | 0.156359 |
| *Thamnolia vermicularis* s. *vermicularis* Sw. Schaer. | 0.000003 | 0.158018 |
| *Trollius* x *apertus* Perfil. ex Igoschina | -0.000016 | 0.158207 |
| *Cephalozia bicuspidata* L. Dumort. | 0.000014 | 0.160226 |
| *Bryum weigelii* Sprengel | -0.000024 | 0.163866 |
| *Trisetum spicatum* L. K. Richt. | 0.000008 | 0.167549 |
| *Lichenomphalia hudsoniana* H.S. Jenn. Redhead et al. | 0.000011 | 0.171463 |
| *Pyrola minor* L. | -0.000014 | 0.173824 |
| *Pohlia drummondii* C. Müll. Andrews | -0.000010 | 0.175445 |
| *Eriophorum russeolum* Fr. ex Hartm. | 0.000004 | 0.180176 |
| *Armeria scabra* Pall. ex Roem.. Schult. | 0.000007 | 0.181572 |
| *Angelica decurrens* Ledeb. B. Fedtsch. | -0.000018 | 0.192128 |
| *Comarum palustre* L. | -0.000006 | 0.195407 |
| *Polytrichastrum alpinum* Hedw. G. L. Sm. | 0.000005 | 0.196601 |
| *Luzula wahlenbergii* Rupr. | -0.000005 | 0.202920 |
| *Dryas octopetala* L. | 0.000005 | 0.203716 |
| *Cladonia amaurocraea* Flörke. Schaer. | 0.000003 | 0.204726 |
| *Oncophorus wahlenbergii* Brid. | 0.000005 | 0.208172 |
| *Plagiothecium denticulatum* Hedw. Schimp. | -0.000021 | 0.212782 |
| *Luzula multiflora* s. *frigida* Buchenau. V.I. Krecz. | -0.000016 | 0.217864 |
| *Sphagnum teres* Schimp. Ångstr. | -0.000013 | 0.219422 |
| *Cetraria odontella* Ach. Ach. | -0.000011 | 0.222798 |
| *Carex vaginata* s. *vaginata* Tausch | 0.000005 | 0.227052 |
| *Minuartia rubella* Wahlenb. Hiern | -0.000016 | 0.236717 |
| *Dicranum bonjeanii* De Not. | 0.000011 | 0.238554 |
| *Hypnum cupressiforme* Hedw. | 0.000012 | 0.239878 |
| *Pertusaria species* | -0.000016 | 0.249797 |
| *Rumex acetosella* s. *arenicola* Y. Mäkinen ex Elven | -0.000006 | 0.260655 |
| *Nephroma species* | -0.000013 | 0.264874 |
| *Plagiomnium medium* Bruch. Schimp. T. Kop. | -0.000009 | 0.268904 |
| *Deschampsia anadyrensis* V.N. Vassil. | -0.000011 | 0.283628 |
| *Cladonia bellidiflora* Ach. Schaer. | -0.000005 | 0.300482 |
| *Warnstorfia pseudostraminea* C. Müll. Tuom.. T. Kop. | -0.000013 | 0.302107 |
| *Carex canescens* s. *canescens*. L. | -0.000014 | 0.306619 |
| *Sphaerophorus globosus* Huds. Vain. | -0.000002 | 0.313372 |
| *Dicranum majus* Turner | -0.000005 | 0.314117 |
| *Cladonia rangiferina* L. Nyl. | -0.000003 | 0.316922 |
| *Brachythecium turgidum* C.J. Hart. Kindb. | -0.000008 | 0.320661 |
| *Sphagnum obtusum* Warnst. | 0.000009 | 0.339299 |
| *Pertusaria oculata* Dicks. Th. Fr. | -0.000013 | 0.341499 |
| *Cladonia stellaris* Opiz. Pouzar. Vezda | -0.000004 | 0.348554 |
| *Bryoria nitidula* Th. Fr. Brodo. Hawksw. | 0.000003 | 0.352866 |
| *Dicranum fuscescens* Turner | 0.000004 | 0.358104 |
| *Cladonia sulphurina* Michx. Fr. | 0.000005 | 0.372763 |
| *Peltigera leucophlebia* Nyl. Gyeln. | -0.000004 | 0.390112 |
| *Erigeron species* | -0.000013 | 0.395990 |
| *Pseudobryum cinclidioides* Huebener. T. Kop. | -0.000007 | 0.397860 |
| *Solorina crocea* L. Ach. | -0.000004 | 0.398048 |
| *Polemonium acutiflorum* Willd. | 0.000002 | 0.405446 |
| *Dicranum species* | 0.000002 | 0.431723 |
| *Salix glauca* L. | 0.000002 | 0.432255 |
| *Parmelia omphalodes* s. lat. L. Ach. | 0.000005 | 0.475461 |
| *Oxyria digyna* L. Hill | 0.000003 | 0.478112 |
| *Sphagnum subsecundum* Nees | 0.000006 | 0.480208 |
| *Plagiothecium berggrenianum* Frisvoll | 0.000005 | 0.489525 |
| *Diphasiastrum alpinum* L. Holub | -0.000008 | 0.491078 |
| *Arctophila fulva* Trin. Andersson | -0.000005 | 0.508723 |
| *Carex brunnescens* Pers. Poir. | -0.000008 | 0.535076 |
| *Cetraria species* | -0.000006 | 0.539227 |
| *Cetraria ericetorum* Opiz | 0.000006 | 0.547883 |
| *Epilobium palustre* L. | 0.000005 | 0.548013 |
| *Scorpidium species* | -0.000008 | 0.556212 |
| *Cladonia stricta* s. lat. Nyl. Nyl. | -0.000005 | 0.562190 |
| *Cardamine bellidifolia* s. *bellidifolia*. L. | 0.000005 | 0.563938 |
| *Cladonia squamosa* s. lat. Hoffm. | -0.000003 | 0.565584 |
| *Prasanthus suecicus* Gottsche. Lindb. | -0.000008 | 0.577818 |
| *Cladonia verticillata* s. lat. Ach. Flot. | -0.000005 | 0.584188 |
| *Ochrolechia androgyna* Hoffm. Arn. | -0.000005 | 0.584969 |
| *Peltigera malacea* Ach. Funck | 0.000003 | 0.586126 |
| *Stereocaulon paschale* L. Hoffm. | 0.000002 | 0.594261 |
| *Koeleria asiatica* Domin | 0.000005 | 0.598074 |
| *Peltigera species* | 0.000003 | 0.623970 |
| *Castilleja lapponica* Gand. ex Rebrist. | -0.000005 | 0.625387 |
| *Abietinella abietina* Hedw. Fleisch. | 0.000005 | 0.627659 |
| *Lophozia savicziae* Schljakov | -0.000006 | 0.633057 |
| *Cetraria aculeata* Schreb. Link. | 0.000002 | 0.649896 |
| *Sphagnum girgensohnii* Russ. | -0.000002 | 0.651957 |
| *Artemisia tilesii* Ledeb. | 0.000002 | 0.660484 |
| *Festuca rubra* L. | -0.000003 | 0.669103 |
| *Cladonia crispata* s. lat. Ach. Flot. | 0.000003 | 0.674985 |
| *Empetrum nigrum* L. | 0.000001 | 0.675389 |
| *Schljakovianthus quadrilobus* Lindb. Konstant. Vilnet | -0.000004 | 0.693195 |
| *Calliergon giganteum* Schimp. Kindb. | 0.000003 | 0.708072 |
| *Bryum caespiticium* Hedw. | 0.000004 | 0.721959 |
| *Stereocaulon species* | -0.000002 | 0.722624 |
| *Peltigera polydactylon* Neck. Hoffm. | 0.000002 | 0.732074 |
| *Conostomum tetragonum* Hedw. Lindb. | -0.000002 | 0.733658 |
| *Sarmenthypnum sarmentosum* Wahlenb. Tuom.. T. Kop. | 0.000003 | 0.734581 |
| *Sibbaldia procumbens* L. | -0.000004 | 0.740753 |
| *Sphagnum angustifolium* Warnst. C. Jens. | -0.000004 | 0.749338 |
| *Pedicularis hirsuta* L. | 0.000001 | 0.755435 |
| *Stellaria palustris* Ehrh. ex Hoffm. | -0.000003 | 0.759902 |
| *Salix arctica* Pall. | -0.000003 | 0.793425 |
| *Cetraria nigricans* Nyl. | -0.000001 | 0.803617 |
| *Cladonia cenotea* Ach. Schaerer | -0.000001 | 0.828046 |
| *Pogonatum dentatum* Menzies ex Brid. Brid. | 0.000001 | 0.831824 |
| *Eriophorum scheuchzeri* Hoppe | 0.000001 | 0.842335 |
| *Silene involucrata* Cham. Schltdl. Bocquet | 0.000002 | 0.851038 |
| *Ranunculus species* | -0.000002 | 0.879684 |
| *Calamagrostis lapponica* Wahlenb. Hartm. | -0.000001 | 0.882769 |
| *Minuartia arctica* Steven ex Ser. Graebn. | 0.000001 | 0.895825 |
| *Cassiope tetragona* s. *tetragona* L. D. Don | 0.000000 | 0.937204 |
| *Lophozia species* | -0.000001 | 0.939779 |
| *Peltigera neckeri* Müll.Arg. | 0.000000 | 0.971777 |
| *Bryum capillare* Hedw. | 0.000000 | 0.976269 |
| *Cladonia macroceras* Flörke. Ahti | 0.000000 | 0.989790 |
