# Supplementary figures and images for: Current and past climate co‐shape community‐level plant species richness in the Western Siberian Arctic
Source: Ecol Evol. 2024 Mar 17;14(3):e11140. doi: 10.1002/ece3.11140 (PMC10944673; doi:10.1002/ece3.11140)

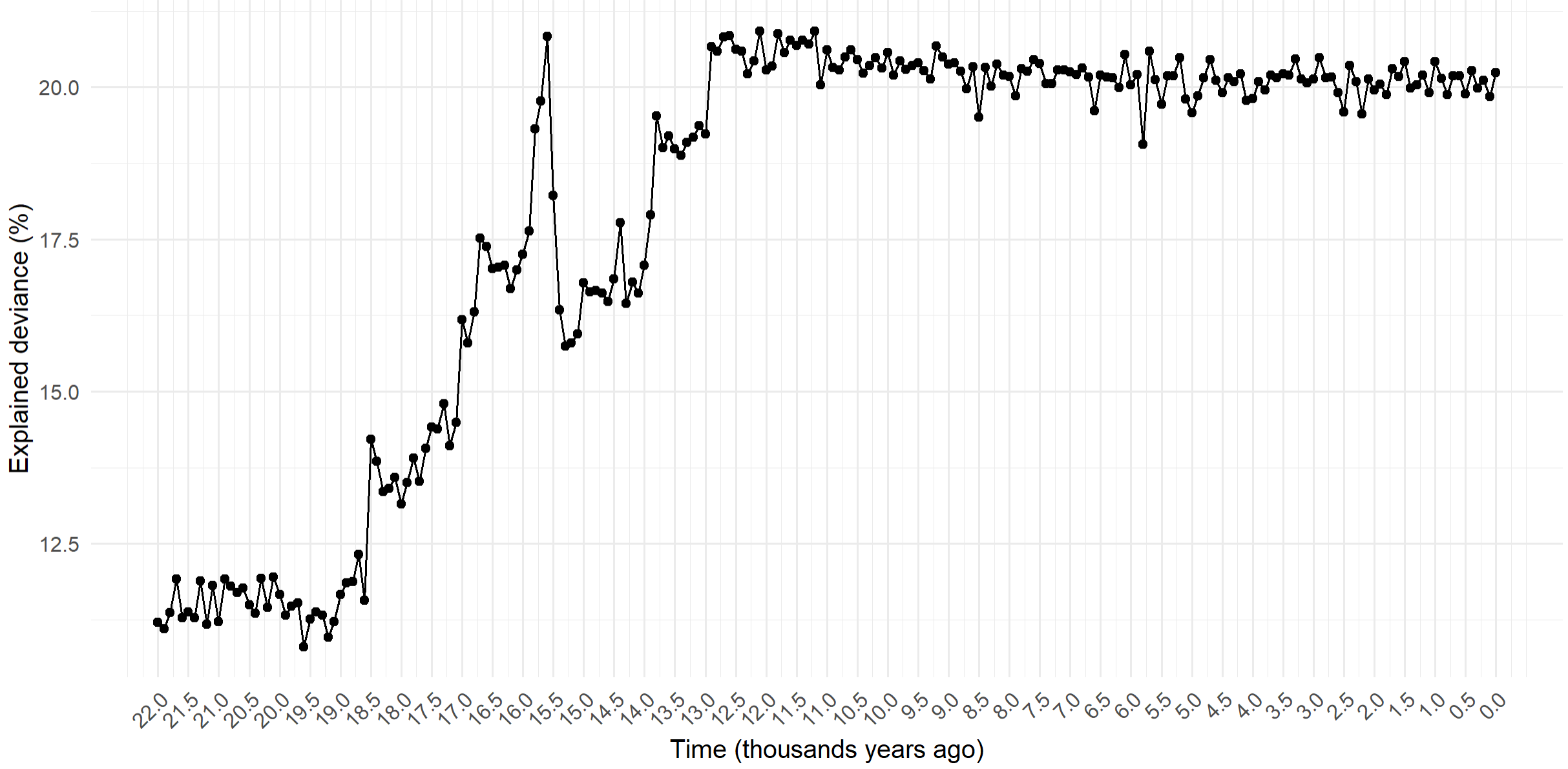

Supplement: Supplementary file 1 — FiguresS1–S6 and Table S1–S3. [file ECE3-14-e11140-s001.zip › 3_ECE3_11140_Appendix_Figure_4.PNG]

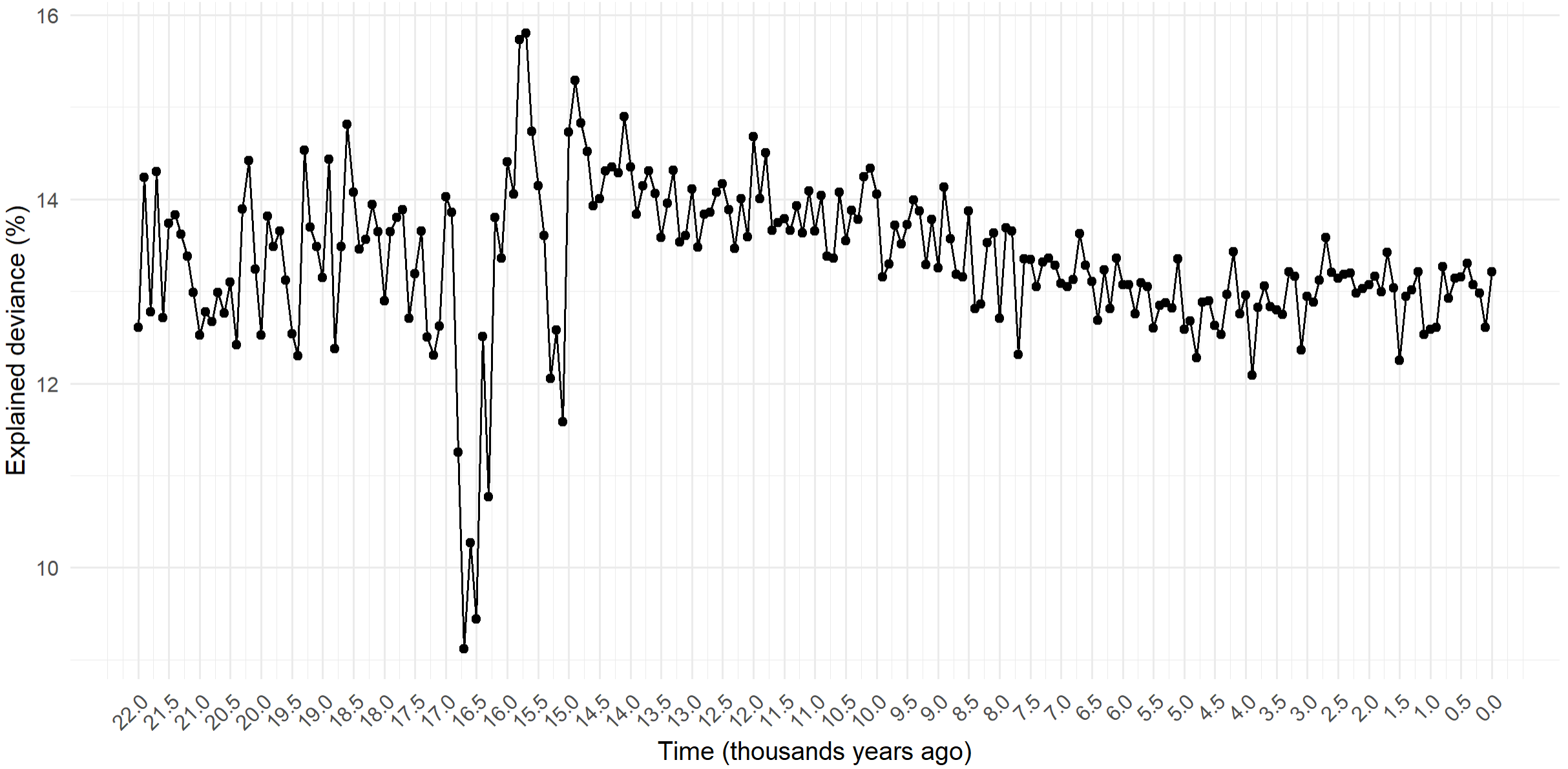

Supplement: Supplementary file 1 — FiguresS1–S6 and Table S1–S3. [file ECE3-14-e11140-s001.zip › 3_ECE3_11140_Appendix_Figure_5.PNG]

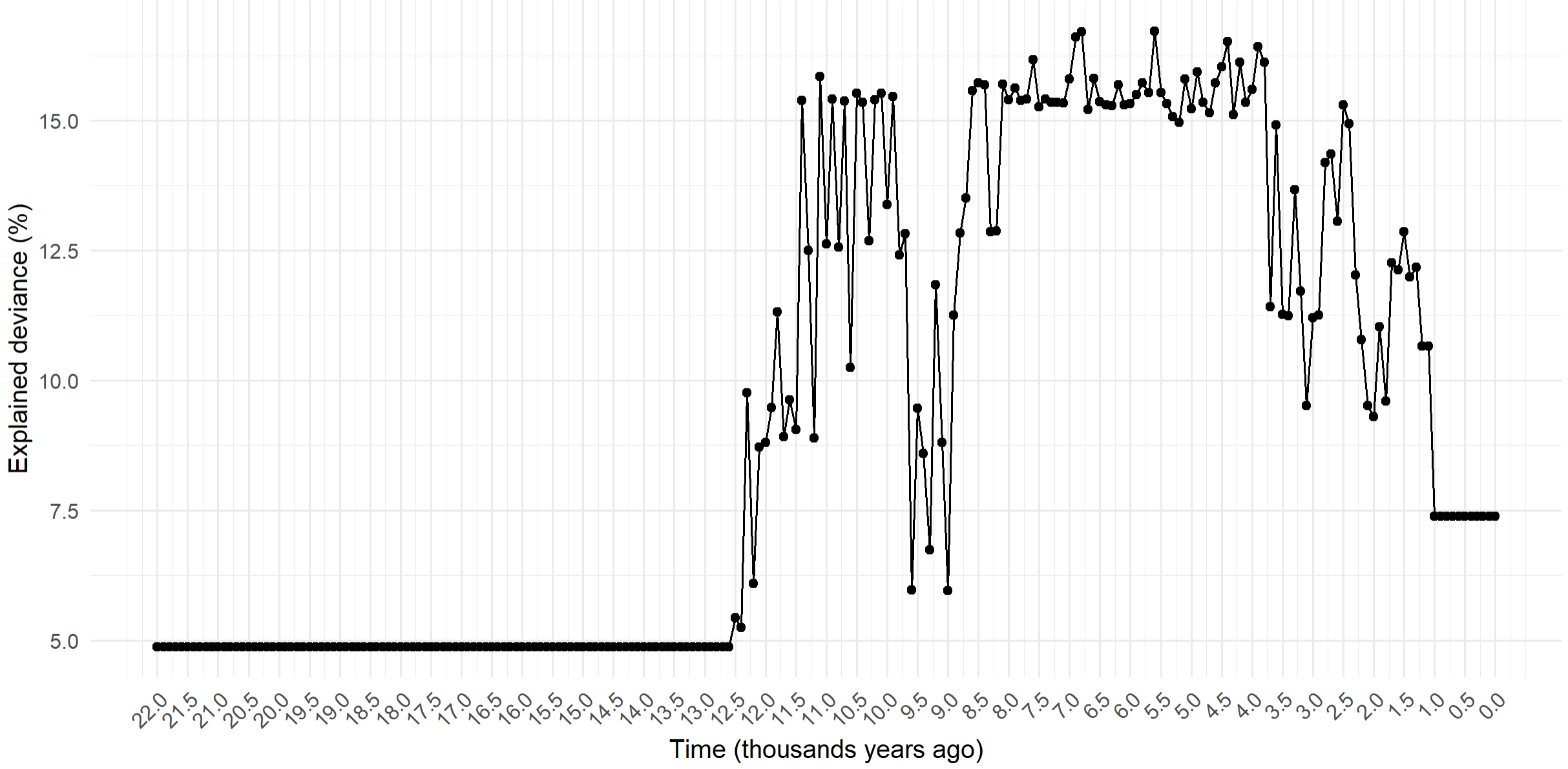

Supplement: Supplementary file 1 — FiguresS1–S6 and Table S1–S3. [file ECE3-14-e11140-s001.zip › 3_ECE3_11140_Appendix_Figure_6.PNG]

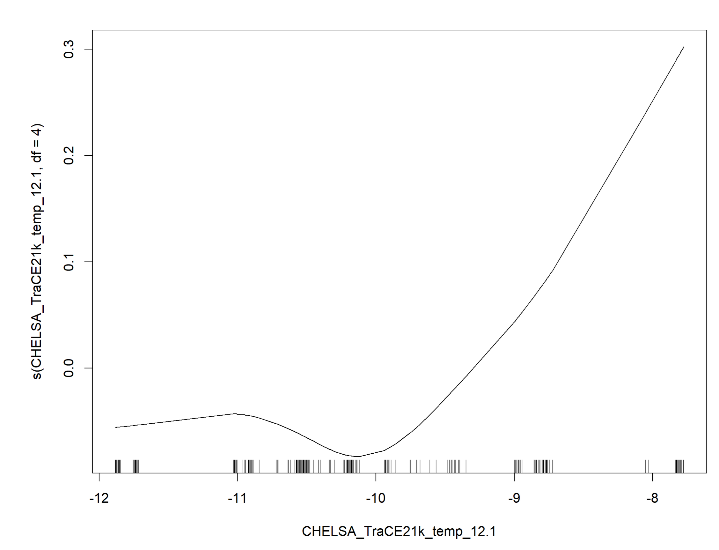

Supplement: Supplementary file 1 — FiguresS1–S6 and Table S1–S3. [file ECE3-14-e11140-s001.zip › ECE3_11140_SE_Fig.1a_temp12.1.png]

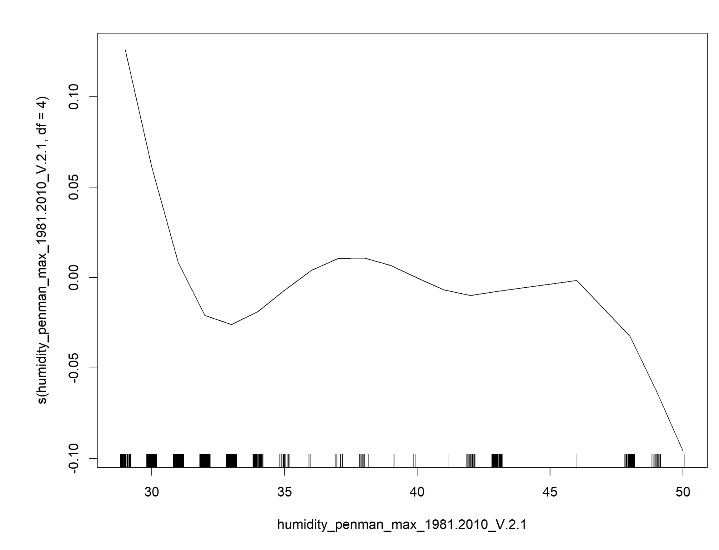

Supplement: Supplementary file 1 — FiguresS1–S6 and Table S1–S3. [file ECE3-14-e11140-s001.zip › ECE3_11140_SE_Fig.1b_penman_max.png]

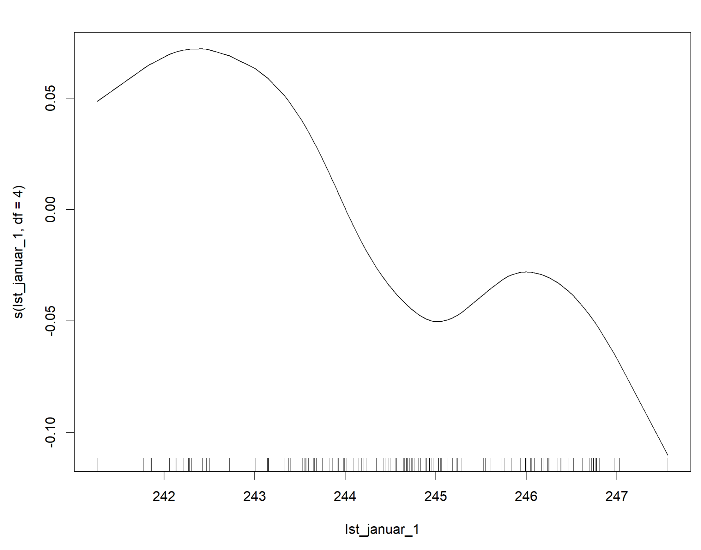

Supplement: Supplementary file 1 — FiguresS1–S6 and Table S1–S3. [file ECE3-14-e11140-s001.zip › ECE3_11140_SE_Fig.1c_januar.png]

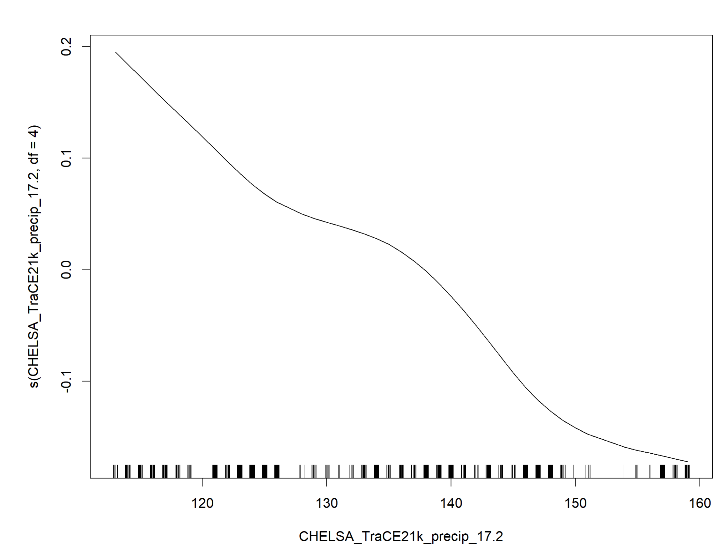

Supplement: Supplementary file 1 — FiguresS1–S6 and Table S1–S3. [file ECE3-14-e11140-s001.zip › ECE3_11140_SE_Fig.1d_precip17.2.png]

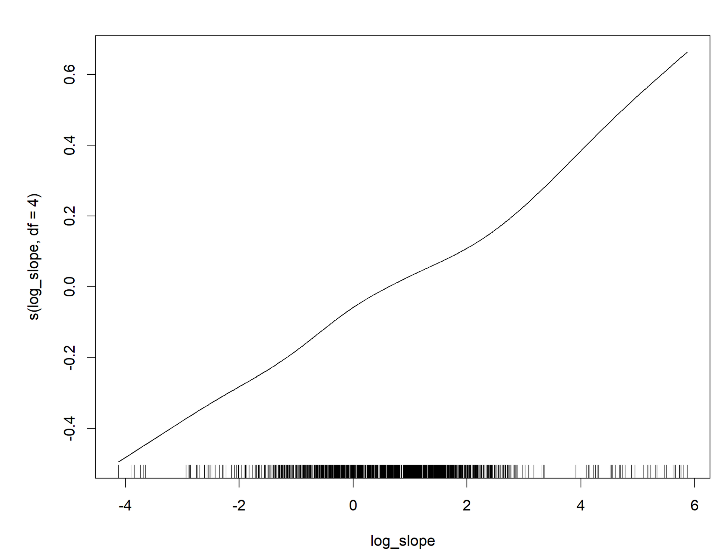

Supplement: Supplementary file 1 — FiguresS1–S6 and Table S1–S3. [file ECE3-14-e11140-s001.zip › ECE3_11140_SE_Fig.1e_slope.png]

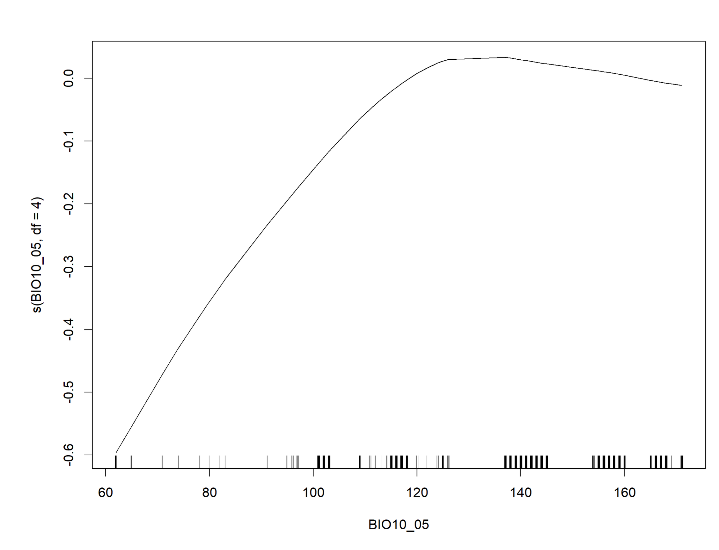

Supplement: Supplementary file 1 — FiguresS1–S6 and Table S1–S3. [file ECE3-14-e11140-s001.zip › ECE3_11140_SE_Fig.1f_bio05.png]

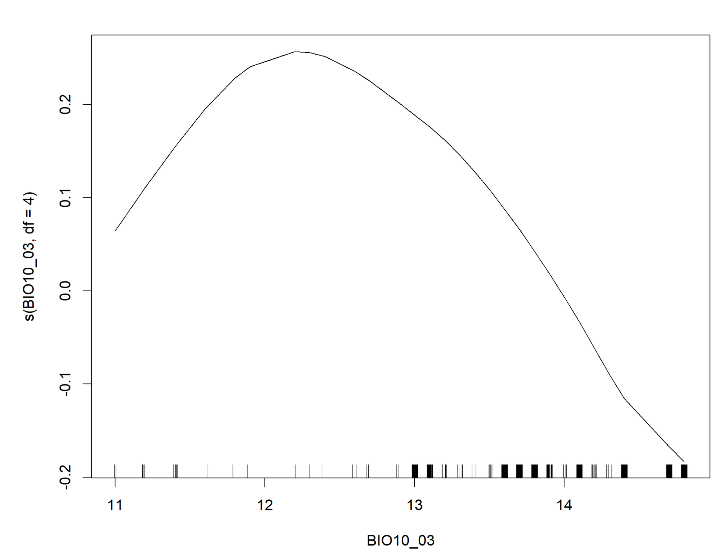

Supplement: Supplementary file 1 — FiguresS1–S6 and Table S1–S3. [file ECE3-14-e11140-s001.zip › ECE3_11140_SE_Fig.1g_bio10_03.png]

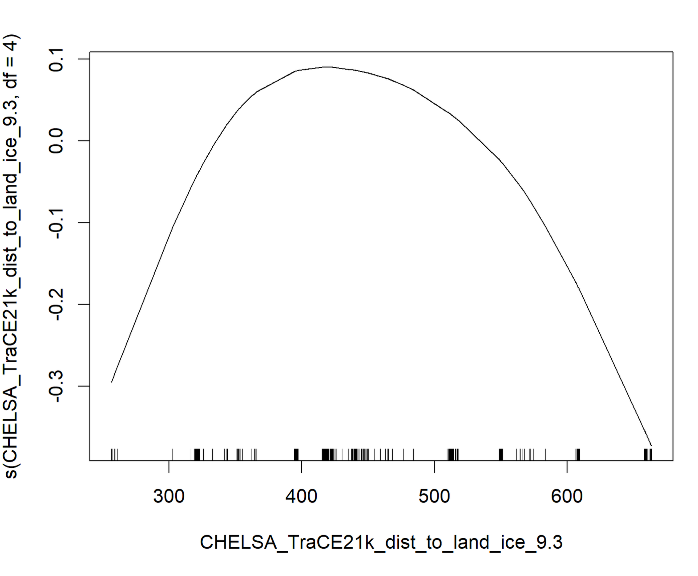

Supplement: Supplementary file 1 — FiguresS1–S6 and Table S1–S3. [file ECE3-14-e11140-s001.zip › ECE3_11140_SE_Fig.1h_landice.png]

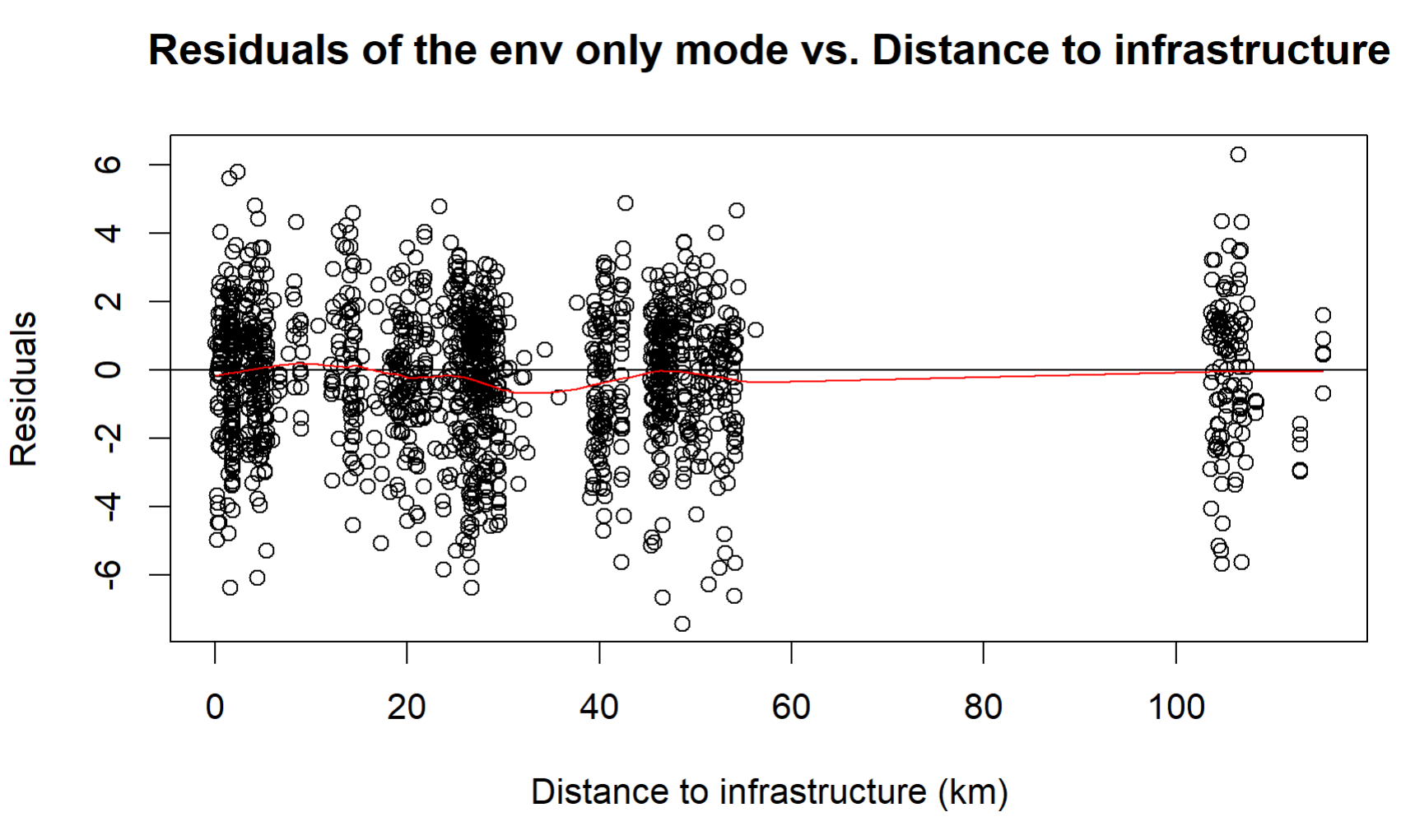

Supplement: Supplementary file 1 — FiguresS1–S6 and Table S1–S3. [file ECE3-14-e11140-s001.zip › ECE3_11140_SE_Fig.2.png]

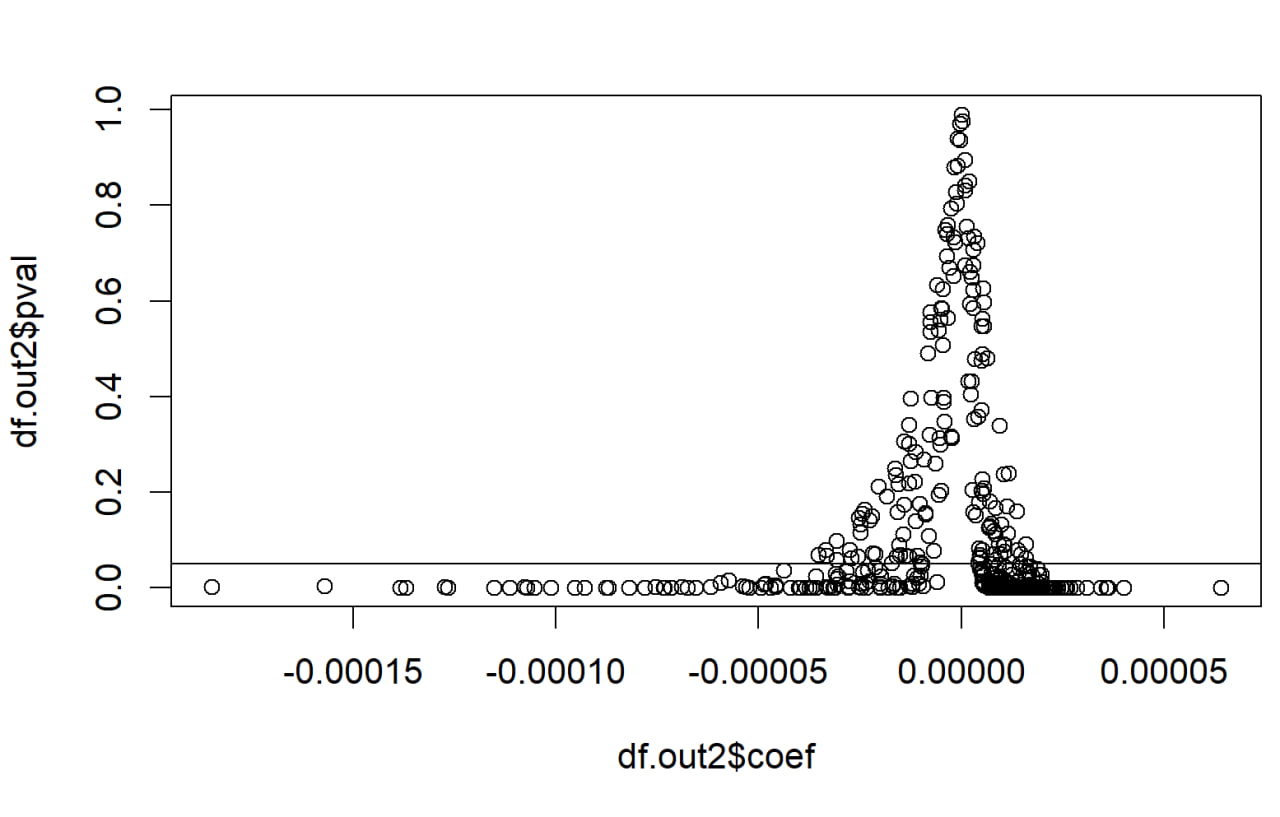

Supplement: Supplementary file 1 — FiguresS1–S6 and Table S1–S3. [file ECE3-14-e11140-s001.zip › ECE3_11140_SE_Fig.3.png]
